# Endothelin-1, over-expressed in SOD1^G93A^ mice, aggravates injury of NSC34-hSOD1G93A cells through complicated molecular mechanism revealed by quantitative proteomics analysis

**DOI:** 10.3389/fncel.2022.1069617

**Published:** 2022-12-01

**Authors:** Yingzhen Zhang, Lin Chen, Zhongzhong Li, Dongxiao Li, Yue Wu, Yansu Guo

**Affiliations:** ^1^Department of Neurology, The Second Hospital of Hebei Medical University, Shijiazhuang, China; ^2^Beijing Geriatric Healthcare Center, Xuanwu Hospital, Capital Medical University, Beijing, China; ^3^Beijing Municipal Geriatric Medical Research Center, Beijing, China

**Keywords:** amyotrophic lateral sclerosis, TgSOD1-G93A, NSC34-hSOD1G93A cells, ET-1, ET-A, ET-B, proteomics, bioinfomatic analysis

## Abstract

Endothelin-1 (ET-1), a secreted signaling peptide, is suggested to be involved in multiple actions in various tissues including the brain, but its role in amyotrophic lateral sclerosis (ALS) remains unknown. In this study, we detected the expression changes as well as the cellular localization of ET-1, endothelin A (ET-A) and endothelin B (ET-B) receptors in spinal cord of transgenic SOD1-G93A (TgSOD1-G93A) mice, which showed that the two ET receptors (ET-Rs) expressed mainly on neurons and decreased as the disease progressed especially ET-B, while ET-1 expression was up-regulated and primarily localized on astrocytes. We then explored the possible mechanisms underlying the effect of ET-1 on cultured NSC34-hSOD1G93A cell model. ET-1 showed toxic effect on motor neurons (MNs), which can be rescued by the selective ET-A receptor antagonist BQ-123 or ET-B receptor antagonist BQ-788, suggesting that clinically used ET-Rs pan-antagonist could be a potential strategy for ALS. Using proteomic analysis, we revealed that 110 proteins were differentially expressed in NSC34-hSOD1G93A cells after ET-1 treatment, of which 54 were up-regulated and 56 were down-regulated. Bioinformatic analysis showed that the differentially expressed proteins (DEPs) were primarily enriched in hippo signaling pathway-multiple species, ABC transporters, ErbB signaling pathway and so on. These results provide further insights on the potential roles of ET-1 in ALS and present a new promising therapeutic target to protect MNs of ALS.

## Introduction

Amyotrophic lateral sclerosis (ALS) is a neurodegenerative disease featured with selective loss of motor neurons (MNs). The symptoms often manifest at first in distal muscles of a single limb and then spread throughout the body, which ultimately cause total paralysis (Maniatis et al., 2019; Masrori and Van, 2020). Although plenty of studies have been performed until now, the mechanisms through which it becomes pervasive, and the processes that initiate ALS pathology remain unclear. Transgenic mice overexpressing the G93A mutation in human SOD1 (hSOD1G93A) (Gurney et al., 1994) was widely used, which can recapitulate the typical phenotype of ALS patients (Beqollari et al., 2016). NSC34 cells transfected with hSOD1G93A (NSC34-hSOD1G93A cells) (Cashman et al., 1992) were also put to use for the reason that they can show decreased viability, lowered proliferation rate, mitochondrial dysfunction, and greater vulnerability to oxidation-induced cell death (Gomes et al., 2008). The above two models are classic for ALS studies.

Endothelin (ET) is a vasoactive peptide, consisting of three secreted ET peptide ligands (ET-1, ET-2 and ET-3). The three isoforms are 21-amino acid cyclic peptides which all contain an N-terminal that determines the affinity for their receptor and a C-terminal which mediates the receptor binding itself (Khimji and Rockey, 2010). Multiple functions of ET were postulated, especially the function of ET-1 in nervous system. For example, ET-1 plays roles in neurotransmission and induces excitation of neurons in the spinal cord and trigeminal system. In addition, ET-1 is implicated in many central nervous system (CNS) pathologies that involve reactive gliosis (Koyama, 2013; Jain et al., 2022). ET-1 can produce biological actions by acting on two types of receptors (ET-A and ET-B) (Arai et al., 1993; Haryono et al., 2022). In brain, high concentrations of ET-1 are generated by neurons, astrocytes and other glial cells and involved in neuronal proliferation, survival, and differentiation (Marola et al., 2022; Ranjan and Gulati, 2022). Meanwhile, both ET-A and ET-B receptors are implicated regulators of homeostatic conditions in the CNS, which adjust the sympathetic nervous system and cerebral blood flow (CBF) (Vidovic et al., 2008). Stimulation of different ET-1 receptors confers different pathogenetic functions, including the increase of free radical generation and subsequent mitochondrial dysfunction (Aliev, 2011), enhancing cerebral perfusion and behavioral results in rats suffered from traumatic brain insult (Kreipke et al., 2011) or resulting in activation of astrocytes proliferation, hypertrophy and cytoskeletal reorganization, eventually astrogliosis (Rogers et al., 2003).

ET-1 was involved in neurologic diseases including Alzheimer’s disease (AD) and multiple sclerosis (MS). Tam et al. (2019) identified that overexpression of astrocytic ET-1 reinforced memory deficits in aged mice or in APP^K670/M671^ mutant mice and induced cofilin rod formation thus producing toxic effects similar to Aβ on neurons. Meanwhile, ET-1-upregulated astrocytes exhibited amyloid secretion after hypoxic/ischemic injury through phosphoinositide 3-kinase (PI3K)/serine/threonine kinase (Akt)-dependent way, revealing the negative role of ET-1 (Hung et al., 2015). ET-1 released from such astrocytes may reach smooth muscle cells of the cerebral microvasculature and reduce cerebrospinal fluid (CBF). The presence of white matter lesions in AD was indeed associated with significantly reduced CBF compared to AD patients without these lesions. Compared to the age-match controls, increased ET-1 was detected in the frontal, temporal and occipital lobes of the post-mortem brain of AD patients with a more significant influence marked in the neurons, reactive astrocytes and cerebral vessel walls (Palmer et al., 2012). Similar to AD, the increasing ET-1 can explain why patients with MS have a reduced CBF in both white and gray matter. Treatment with the mixed ET receptor antagonist bosentan in MS patients restored CBF to levels found in controls. The possible source of this ET-1 production are reactive astrocytes in focal MS lesions that express high levels of ET-1, however, resting astrocytes in human brain visually do not exhibit ET-1 immunoreactivity (D’haeseleer et al., 2013). Besides, ET-1 signals indirectly inhibited oligodendrocyte progenitor cells (OPC) differentiation and remyelination through ET-B receptor in MS injury model (Hammond et al., 2014, 2015).

Recently, Ranno et al. (2014) found that expression of ET-1 was increased in the spinal cord reactive astrocytes of ALS mice and sporadic ALS patients, and its toxic effect was proved on cultured motor neurons through a series of *in vitro* experiments (Ranno et al., 2014). In addition, microarray analysis of fibroblasts from familial ALS patients with C9orf72p mutations identified ET-1 as one of the core genes in the protein-protein interaction network (Kotni et al., 2016). However, the exact mechanism of ET-1 is still unknown and there is a great interest in studying the physiological localization and pathological changes of ET-1 related signaling elements as well as its underlying molecular pathways in ALS.

In this study, we investigated the expression changes of ET-1/ET-Rs in the SOD1-G93A mouse model of ALS. Additionally, the effects of ET-1 on NSC34-hSOD1G93A cells were also assessed and potential molecular mechanism was explored using the strategy of integrated quantitative proteomics. Our findings help us better understand the detailed effect of ET-1 and give us more consideration to the possible neuroprotective strategies against ALS.

## Materials and methods

### Animals

Transgenic human SOD1-G93A mice and their non-transgenic (NTG) littermates were produced by breeding female B6SJLF1 hybrids with male hemizygous carriers [B6SJL-Tg (SOD1-G93A) 1Gur/J], which were purchased from the Jackson Laboratory (Bar Harbor, ME, USA). The SOD1-G93A mouse genotyping was performed as described previously (Li et al., 2017). All animals were group-housed (4–5 mice/cage) under identical conditions (12 h light/dark cycle with free access to food and water). Since the clinical phenotype of SOD1-G93A mice, characterized by an adult onset of motor symptoms in the hind limbs, was first manifested around 12 weeks of age, and pathological changes in spinal cord begin to be observed around 60 days which progresses continuously to end stage at 17–20 weeks (Weydt et al., 2003; Ludolph et al., 2007), SOD1-G93A mice at three predefined stages were used in this study: (1) Pre-symptomatic: 40 days, postnatal, no signs of motor deficit; (2) Onset: 80 days, postnatal, initial signs of motor deficit; (3) End: animals can no longer right themselves 30 s after being placed on their backs or sides. The experiments were conducted in accordance with the regulations of laboratory animal management promulgated by the Ministry of Science and Technology of the People’s Republic of China, and approved by the Ethics Committee of the Second Hospital of Hebei Medical University. All mice were narcotized under 1.0–3.0% (vol/vol) isoflurane (RWD, Shenzhen, China), and we made all efforts to minimize suffering.

### Neuroblastoma/spinal cord hybrid cell line cells culture and endothelin-1 intervention

The mouse neuroblastoma/spinal cord hybrid cell line (NSC34) is a motoneuron-like cell line through the fusion of embryonic mouse spinal cord motor neurons and mouse neuroblastoma cells. NSC34 cells stably transfected with GFP-empty vector (E), GFP-human SOD1 wild type (hSOD1WT), or GFP-human SOD1G93A (hSOD1G93A) were established as previously described in our laboratory (Bai et al., 2021). The cell lines were incubated in Dulbecco’s modified Eagle’s medium (DMEM), including 10% heat-inactivated fetal bovine serum (FBS) and 1% penicillin streptomycin. To maintain the stable cell lines, 0.2 mg/mL Geneticin 418 sulfate (G418) was used. Cells were cultured at 37^°^C under a 5% CO_2_ humidified atmosphere, refreshing the medium every 1–2 days. When 80–90% confluence was reached, cells were passaged using a 0.25% trypsin/EDTA solution for subsequent experiments. The cells were starved in serum-free DMEM for 24 h, and then incubated in medium containing ET-1 (1 nM, 10 nM, 100 nM, 1,000 nM) for 24 h or with a fixed 100 nM ET-1 concentration for different lengths of time (12 h, 24 h, 48 h). For rescue experiments, 100 nM ET-1 in combination with BQ123 or BQ788 was added to the cells and then incubated for 24 h.

### Cell counting kit-8 assay

The measurement of cell viability was performed by a cell counting kit-8 (CCK-8) assay (BOSTER Biological Technology Co., Ltd., Wuhan, China) (Liu et al., 2014). In short, after a 24 h exposure to the respect treatment, we added 10 μL of CCK-8 solution to each well and incubated the cells at 37^°^C for 1 h in the dark. Then, a microplate reader was used to measure the absorbance at a wavelength of 450 nm (Tecan Spark 10 μM, Switzerland). All experiments were performed at least three times independently.

### Immunohistochemistry

The SOD1-G93A mice and NTG mice at different disease stages were perfused with 0.1 M ice-cold phosphate buffered saline (PBS), followed by perfusion with 4% paraformaldehyde (PFA). The spinal cords were collected and lumbar segments (L3-5) were post-fixed in 4% PFA/PBS at 4^°^C for 24 h. The following day, the tissues were cryoprotected in 30% sucrose at 4^°^C overnight and then embedded in optimum cutting temperature (OCT) compound. The lumbar segments (L3-5) were sliced into coronal sections (25 μm thick) with a Leica cryostat (CM1850). Sections were treated with 3% hydrogen peroxide (H_2_O_2_) for 10 min and then washed in PBS with gentle agitation for 10 min. Afterward, the sections were permeabilized/blocked with 5% goat serum and 0.3% Triton X-100 in PBS and then co-immunostained for primary antibodies diluted in blocking solution at 4^°^C overnight. The primary antibodies include: rabbit anti-Endothelin-1(Abbiotec, 250633, 1:100), rabbit anti-ETA (Bioss, bs-1757R, 1:300) and rabbit anti-ETB (Abcam, ab117529, 1:500). After incubation, the tissues were washed in PBST (0.2% Tween 20 in PBS) three times, and then incubated with a corresponding biotin-conjugated secondary antibody (1:1,000). Next, after washing three times in PBS, incubating in Vectastain ABC Reagent (Vector Laboratories, Burlingame, CA, USA, PK-6100) for 40 min, color development was performed to the sections by the immPACT DAB Peroxidase Substrate Kit (Vector, SK-4105). At last, the slices were loaded onto slides and dried appropriately. The slides were soaked in anhydrous ethanol for 5 min, xylene for 10 min and finally sealed with Permount TM Mounting Medium (ZSGB-BIO, ZLI-9516). All images were photographed under an Olympus BX51 microscope equipped with a DP72 digital camera system (Olympus, Tokyo, Japan).

### Immunofluorescence

For immunofluorescence, spinal cord sections (25 μm thick) were pretreated with 1% Triton X-100 in PBS for 30 min, and blocked with solution containing 5% donkey serum, 0.3% Triton X-100 and 0.2% dry milk in PBS for 30 min. The sections were then incubated with primary antibodies in blocking buffer overnight at 4^°^C. Primary antibodies include goat anti Iba-1 (Wako, 019-19741, 1:250), mouse anti-GFAP (Millipore, MAB360, 1:400), mouse anti-NeuN (Millipore, MAB377, 1:100), mouse anti-APC (Millipore, OP80, 1:400), rabbit anti-SOD1 (Immunoway, YT4364, 1:100), rabbit anti-cFOS (Abcam, ab222699, 1:100), rabbit anti-MAP1B (Proteintech, 21633-1-AP, 1:100), mouse anti-SMI32 (Biolegend, 801701, 1:100), rabbit anti-GFP (Life tech, G10362, 1:100), rabbit anti-Endothelin-1 (Abbiotec, 250633, 1:100), rabbit anti-ET-A (Bioss, bs-1757R, 1:300) and rabbit anti-ET-B (Abcam, ab117529, 1:500). Thereafter, the sections were washed for 30 min followed by incubation with the appropriate secondary antibody (Alexa Fluor 488, 594 or 647, Invitrogen) at room temperature for 2 h. After further rinsing in PBS for 30 min, sections were stained with mounting medium containing DAPI (VECTOR, VECTASHIELD H-1200) and sealed with nail polish.

Quantitative assessment of motor neuron labeling was performed as follows: Three representative images of the lumbar ventral horns from each tested animal were used for quantitative measurements of motor neurons. Motor neurons were defined according to the following criteria: (1) neurons were located in the anterior horn of the spinal cord and were NeuN^+^ and (2) neurons with a diameter of 20 μm or larger had a distinct nucleolus (Manabe et al., 2003). The images from immunofluorescence staining were captured using an Olympus FV1000 confocal microscopy or Zeiss Vert. A1, German confocal microscopy. The numbers of motor neurons in the ventral horn area and quantification of fluorescence intensity were analyzed using ImageJ software (Java 8, National Institutes of Health, USA) (Jensen, 2013).

### Western blotting

Total protein from frozen tissues was extracted using a protein extraction kit and phenylmethylsulfonyl fluoride protease inhibitors (Beijing Solarbio Science and Technology Co., Ltd., China). 30 μg of protein lysates from each sample were electrophoresed through a 10% or 12% SDS–PAGE and then transferred onto polyvinylidene fluoride (PVDF) membranes. The membranes were blocked with 5% non-fat milk, and, respectively incubated with primary antibodies overnight at 4^°^C, including rabbit anti-SOD1 (Immunoway, YT4364, 1:1,000), rabbit anti-GFP (Life tech, G10362, 1:1,000), rabbit anti-Endothelin-1 (Abbiotec, 250633, 1:300), rabbit anti-ET-A (Bioss, bs-1757R, 1:1,000), rabbit anti-ET-B (Abcam, ab117529, 1:4,000), and rabbit anti-GAPDH (Proteintech, 10494-1-AP, 1:5,000). After incubation, the membranes were washed three times (each for 10 min), and incubated with relevant fluorescence-conjugated secondary antibodies for 2 h at room temperature. Finally, the membranes were detected by an Odyssey Infrared Imaging System (LI-COR, Lincoln, NE, USA), and quantitatively analyzed by ImageJ (Java 8, National Institutes of Health, USA).

### LC-MS/MS analysis

The detailed experimental methods for sample preparation before LC-MS/MS analysis can be found in Supplementary Files. For whole-cell proteome analysis, the tryptic peptides were loaded on an EASY-nLC1200 UPLC system (ThermoFisher Scientific). Tryptic peptides were dissolved in solvent A (0.1% formic acid, 2% acetonitrile/in water), separated with solvent B (0.1% formic acid in 90% acetonitrile) *via* a gradient from 8 to 23% (0–68 min), 23–32% (68–82 min) and climbing to 32–80% (82–86 min) then holding at 80% (86–90 min). Separation was analyzed in Orbitrap Exploris™ 480 (Thermo Fisher Scientific) using a nano-electrospray ion source. The electrospray voltage applied was 2.3 kV and the full MS scan range was set from 400 to 1,200 m/z. Then, more than 25 most abundant precursors were selected for further MS/MS analyses by 20 s dynamic exclusion. The HCD fragmentation was operated at normalized collision energy (NCE) of 27%. The resolution for fragments were 15,000 in the Orbitrap and fixed first mass was set as 110 m/z. Automatic gain control (AGC) target was set at 100%, along with an intensity threshold of 2E4 and a maximum injection time of Auto.

### Database search

The resulting MS/MS data were processed by Proteome Discoverer (v2.4.1.15) search engine (v.1.6.15.0). Tandem mass spectra were searched against the Mus musculus SwissProt database (20,387 entries) and concatenated with reverse decoy database. The detailed parameter settings are shown in Supplementary Files.

### Bioinformatics analysis-protein function enrichment and cluster analysis

To further analyze hierarchical clustering based on functional classification of differentially expressed proteins (such as: GO, Domain, Pathway), all the categories obtained after enrichment along with their *P*-values were collated, and those categories that were at least enriched in one of the clusters with *P* < 0.05 were filtered. We transformed the filtered *P*-value matrix by the function × = –log10 (*P*-value). At last, these × values were z-transformed for each functional category, and then clustered by one-way hierarchical clustering (Euclidean distance, average linkage clustering) in Genesis. A heat map with the “heatmap.2” function from the “gplots” R-package can visualize the cluster membership finally.

### Parallel reaction monitoring analysis

Parallel reaction monitoring (PRM), a technique based on high-resolution and high-precision mass spectrometers (such as Q Exactive™ Plus) can screen the remaining samples of LC-MS/MS proteome for further verification. The sample was slowly added to the final concentration of 20% v/v TCA to precipitate protein, then vortexed to mix and incubated for 2 h at 4^°^C. The precipitate was collected by centrifugation at 4,500 g for 5 min at 4^°^C. The precipitated protein was washed with pre-cooled acetone for 3 times and dried for 2 h. The protein sample was then redissolved in 200 mM TEAB and ultrasonically dispersed. Trypsin was added at 1:50 trypsin-to-protein mass ratio for the first digestion overnight. The sample was reduced with 5 mM dithiothreitol for 30 min at 56^°^C and alkylated with 11 mM iodoacetamide for 15 min at room temperature in darkness. The tryptic peptides were dissolved in 0.1% formic acid (solvent A), and were mounted to a home-made reversed-phase analytical column directly, with a gradient of 6–20% solvent B (0–40 min), then 23–35% (14 min), 20–30% (40–52 min), 30–80% (52–56 min) then 80% (56–60 min) on an EASY-nLC1000 UPLC system using the flow rate of 500 nL/min constantly. Later, risk assessment was performed in the proteins with fold change no less than 1.2 and *P* < 0.05, according to peptide hits, coverage, signal strength and number of second order maps. Risk was divided into high, medium and low risk of three gradients. After grading the peptide residues, they were ionized into the NSI ion source and then analyzed by Q ExactiveTM Plus mass spectrometry. The resulting MS data were processed using Skyline (v.21.1). Each protein in the experiment applied one, two or more unique peptides for quantitative analysis (Chai et al., 2020). Additional Methods showed the full details in Supplementary Files.

### Statistical methods and analyses

Unpaired *t*-test was used to calculate significant differences between two groups and one-way ANOVA was used to assess significant differences within multiple groups. Data were analyzed by SPSS-23 software (IBM SPSS Statistics 23, USA) and GraphPad Prism software (5.0.0; GraphPad Software, USA) were applied to graphing. Statistical significance was defined as *P* < 0.05. Data were expressed as the mean ± SEM.

## Results

### Expression changes of endothelin-1 and ET-A/B receptors in the lumbar spinal cord of SOD1-G93A mice

**FIGURE 1:**
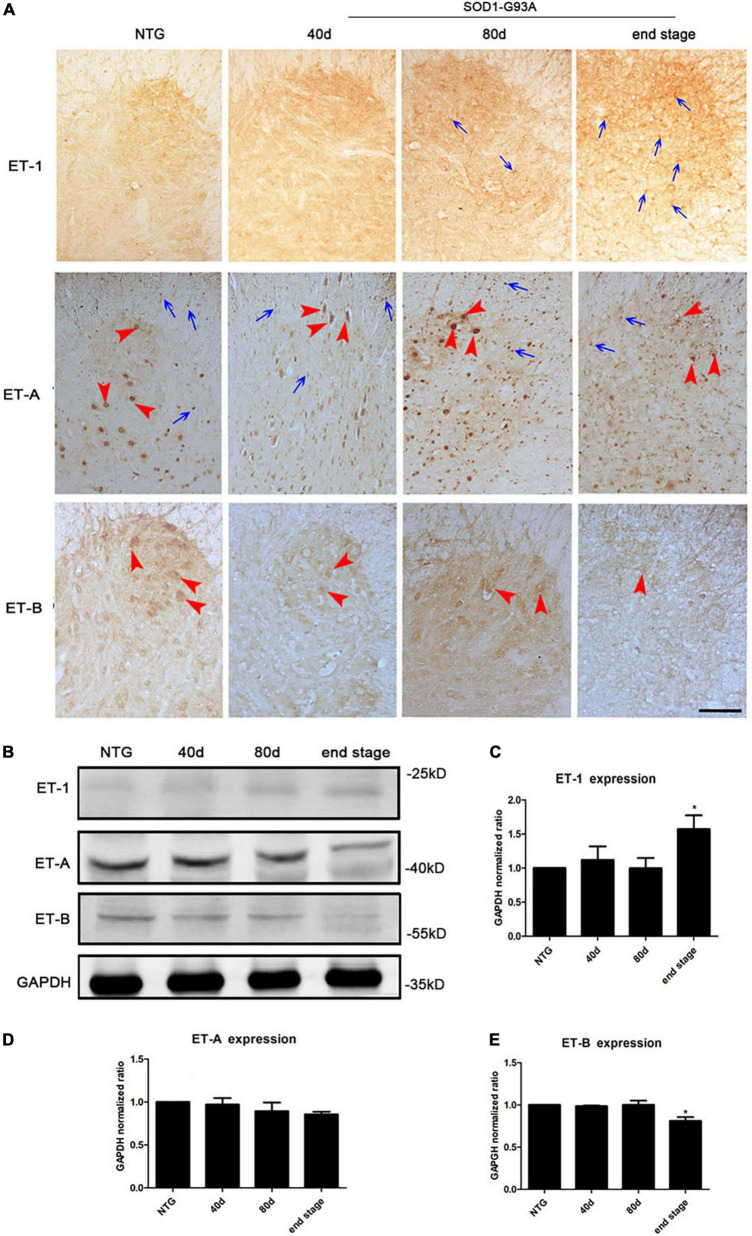
Expression changes of ET-1, ET-A, and ET-B receptors at the end stage of SOD1-G93A mice. **(A)** Immunohistochemical detection of ET-1, ET-A, and ET-B receptors in the lumbar spinal cord of non-transgenic (NTG) mice and SOD1-G93A transgenic mice at 40 days (d), 80 days (d) and end stage. **(B)** Western blot analysis of ET-1, ET-A, and ET-B receptor levels in the lumbar spinal cord of NTG mice and SOD1-G93A transgenic mice at 40d, 80d and end stage. **(C–E)** Quantitative analysis of B. Statistical significance was assessed by one-way ANOVA followed by LSD-*t* test. Data represent the mean ± SEM. *P* (ET-1, end stage vs. NTG) = 0.037; *P* (ET-A, end stage vs. NTG) = 0.159; *P* (ET-B, end stage vs. NTG) = 0.004. *n* = 3. *P < 0.05. The arrows indicate glial cells, and the arrowheads indicate neurons. Bar = 100 μm.

To evaluate whether ET-1/ET-Rs signaling axis prompt neuron degeneration in ALS, we first observed the expression of ET-1, ET-A, and ET-B receptors in the spinal cords of SOD1-G93A mice at different disease stages using immunohistochemistry (Figure 1). An obvious increase of ET-1 positive cells, which were clearly glia-like cells (Figure 1A, arrows), was observed in the lumbar spinal cord of SOD1-G93A mice, especially at the end stage of disease. In NTG mice, ET-A receptor was observed mainly in nucleus, both neurons (Figure 1A, arrowheads) and glial cells (Figure 1A, arrows). With disease progression, ET-A-positive motor neurons reduced, while the total number of ET-A-positive nuclei increased. In addition, we found that ET-B receptor was mainly located in the cytoplasm of neurons (Figure 1A, arrowheads), and an obvious decrease of ET-B expression was observed with disease progression (Figure 1A). Consistent with morphological findings, western blotting results revealed an increase of ET-1 expression and decrease of two ET-Rs expression with disease progression (Figure 1B). Apparently, significant expression changes of ET-1 and ET-B receptor were observed at the end stage of SOD1-G93A mice (Figures 1C,E). For ET-A expression, although a decreasing trend was observed, statistically significant difference was not reached even at the end stage (Figure 1D). Together, above-mentioned results indicate that both glial cells and neurons play a vital role in ET-1/ET-Rs pathway thus contributing to ALS pathogenesis.

### Cellular localization of endothelin-1, endothelin A and endothelin B receptors in the lumbar spinal cord of SOD1-G93A mice

**FIGURE 2:**
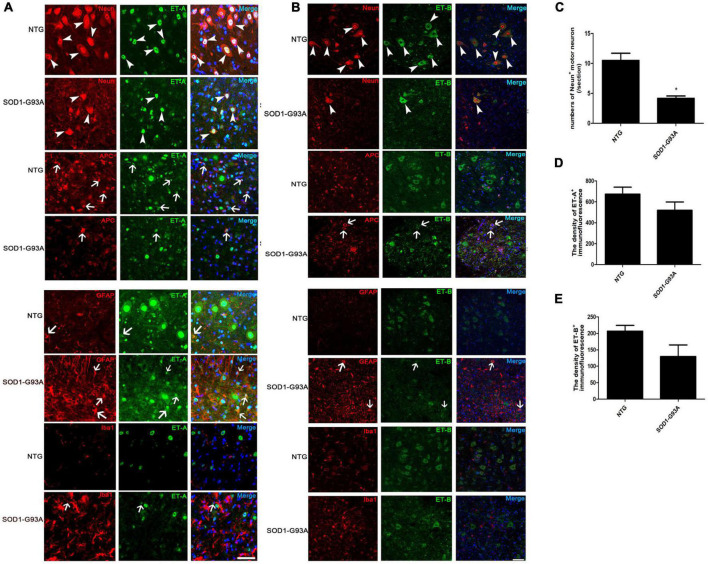
Cellular localization of ET-A and ET-B in the lumbar spinal cord of non-transgenic (NTG) mice and SOD1-G93A transgenic mice at end stage. **(A,B)** are photographed by Olympus FV1000 confocal microscopy, **(C,D)** are photographed by Zeiss Vert. A1, German confocal microscopy. **(A)** Colocalization of ET-A (green) with NeuN (red) in MNs and APC (red) in mature oligodendrocytes of NTG and SOD1-G93A mice; ET-A (green) colocalization with GFAP (red) in astrocytes and Iba1 (red) in microglia of NTG and SOD1-G93A mice. **(B)** Colocalization of ET-B (green) with NeuN (red) in MNs and APC (red) in mature oligodendrocytes of NTG and SOD1-G93A mice; ET-B (green) colocalization with GFAP (red) in astrocytes and Iba1 (red) in microglia, respectively, in the lumbar spinal cord of NTG and SOD1-G93A mice. **(C–E)** The statistical analysis of NeuN^+^ motor neuron numbers, and the fluorescence intensity of ET-A, ET-B in the NeuN/ET-A or NeuN/ET-B co-labeled sections. Data represent the mean ± SEM, statistical significance was assessed by Unpaired *t*-test. *P < 0.05. The arrows indicate glial cells, and the arrowheads indicate neurons. Bar = 50 μm.

To further test the cellular localization of ET-1 and ET-Rs, we then performed double immunofluorescence staining. ET-1 positive cells were mainly observed in proliferating GFAP-positive astrocytes in lumbar spinal cord of ALS mice (Supplementary Figure 1), which was in line with the previous opinions (D’haeseleer et al., 2013; Hostenbach et al., 2016). In normal spinal cord of NTG mice, ET-A expression was primarily located in the nucleus of neurons and glial cells, especially motor neurons. In the spinal cord of ALS mice at end stage, ET-A-positive motor neurons decreased obviously (Figure 2A, arrowheads). However, obvious expression of ET-A was identified in mature oligodendrocytes (APC-positive) (Figure 2A, arrows). Furthermore, just as reported (Ranno et al., 2014), increased ET-A expression was also notably observed on proliferating astrocytes in SOD1-G93A mice compared with NTG mice. In addition, certain ET-A positive microglia was also observed in the spinal cord of SOD1-G93A mice (Figure 2A, arrows).

Similarly, we detected the cellular localization of ET-B and found that ET-B was expressed primarily in the cytoplasm of motor neurons, which was in line with the previous study (Michinaga et al., 2013; Figure 2B, arrowheads). Sparse expression of ET-B in APC was also observed in the lumbar spinal cords of SOD1-G93A mice (Figure 2B, arrows). Interestingly, colocalization of ET-B with GFAP-positive astrocytes was occasionally seen in SOD1-G93A mice (Figure 2B, arrows) and no obvious colocalization of ET-B with Iba1-positive microglia was detected in our experiments either in SOD1-G93A mice or NTG mice (Figure 2B).

Since motor neurons are the main cells expressing both ET-A and ET-B receptors in the normal NTG spinal cord and they degenerate and loss with disease progression, we further ask whether the decrease in ET-A and ET-B expression reflects the general loss of motor neurons. Quantitative analysis showed that the loss of NeuN^+^ motor neurons was parallel with the reduce of both ET-A and ET-B expressions in the spinal cord of SOD1-G93A mice at end stage (Figures 2C–E), which to a great extent explains that the reduce of ET-A and ET-B is largely due to the degeneration of motor neurons in SOD1-G93A mice. However, expression of ET-A and ET-B in proliferating glial cells may partly compensate this reduce.

### Identification of the motor neuronal cell model of amyotrophic lateral sclerosis and its expression of ET receptors

**FIGURE 3:**
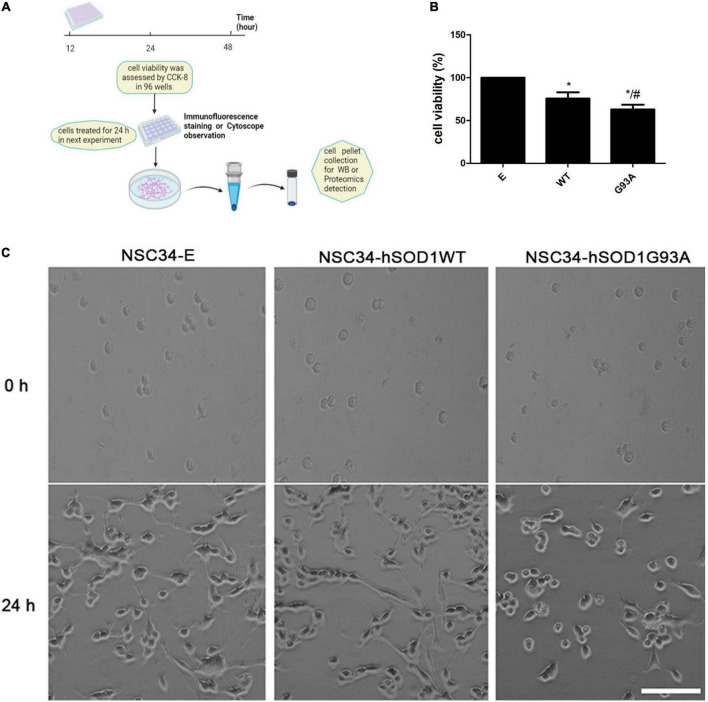
NSC34-hSODG93A cells as the *in vitro* model of ALS. **(A)** Schematic overview of cell experiments. **(B)** Cell viability of NSC34-E, NSC34-hSOD1WT and NSC34-hSOD1G93A cells. **(C)** Cells were seeded in culture dishes, differentiated for additional 24 h and photographed by phase contrast microscopy. Data represent the mean ± SEM, statistical significance was assessed by one-way ANOVA followed by LSD-*t* test. *P < 0.05, *vs*. NSC34-E cells; ^#^*P* < 0.05, NSC34-hSOD1WT cells *vs*. NSC34-hSOD1G93A cells. Bar = 100 μm.

Based on the above results, we proposed that astrocytic ET-1 might have an effect on motor neurons through ET-Rs. To assess the role of ET-1 on motor neurons, we used NSC34 cells stably expressing human SOD1, either wildtype (NSC34-hSOD1WT) or G93A mutated form (NSC34-hSOD1G93A), and NSC34 cells expressing only GFP (NSC34-E) was used as control and conducted a series of experiments (Figure 3A). In order to avoid interference of GFP fluorescence at channel 488, the appropriate secondary antibody (Alexa Fluor 647 or 594, Invitrogen) was used in the cell model.

First, reliability of the cell line model of ALS was confirmed. Western blotting showed that both NSC34-hSOD1 and NSC34-hSOD1G93A expressed GFP-human SOD1 (GFP-hSOD1) fusion proteins, while only GFP was observed in NSC34-E cells (Supplementary Figure 2). Successful transfection was further confirmed by tracing GFP-positive NSC34 cells through immunofluorescence staining for GFP and DAPI. The proportion of GFP-positive NSC34 cells is on average 61 ± 3% (NSC34-E), 66 ± 4% (NSC34-hSOD1WT), and 55 ± 9% (NSC34-hSOD1G93A) (Supplementary Figure 2). CCK-8 assay showed significantly lower cell viability in NSC34-hSOD1 cells, especially NSC34-hSOD1G93A cells compared with NSC34-E cells (Figure 3B). In addition, NSC34-hSOD1G93A cells displayed worse morphological differentiation characterized by less extending neurites which were clearly distinguishable after 24 h compared with NSC34-hSOD1WT or NSC34-E cells (Figure 3C).

**FIGURE 4:**
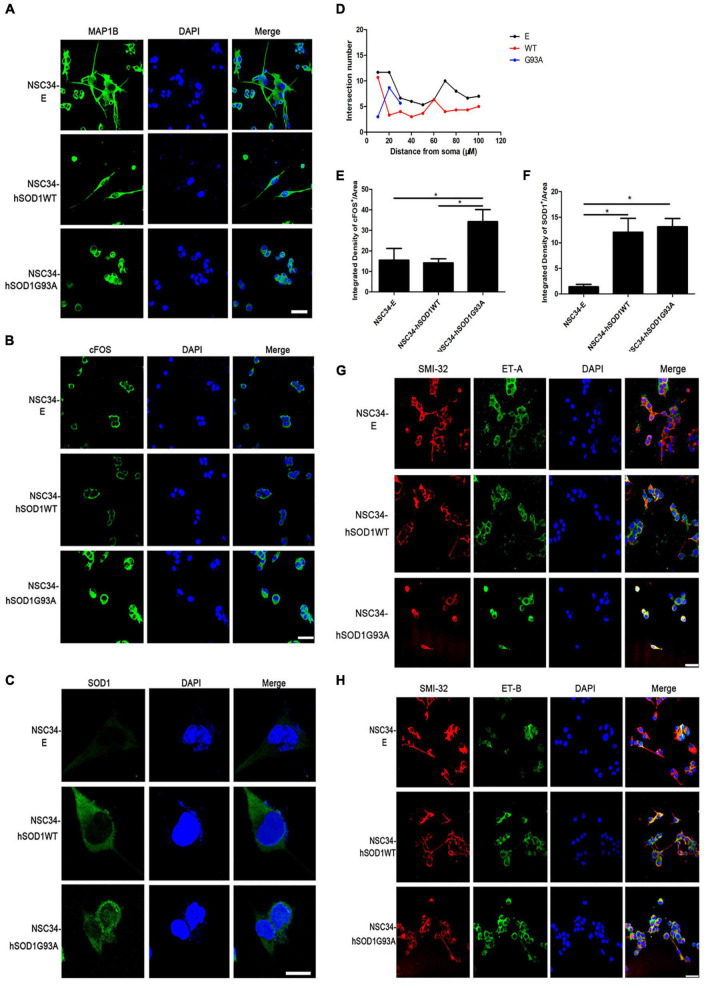
NSC34-hSOD1G93A cells display a series of injured features and ET-A and ET-B receptors are both expressed in the cell model. **(A)** Immunofluorescence labeling of MAP1B showing different neurites of the three cell groups (NSC34-E, NSC34-hSOD1WT and NSC34-hSOD1G93A). **(B)** Immunofluorescence staining indicates increased cFOS expression in NSC34-hSOD1G93A cells. **(C)** Immunofluorescence staining shows hSOD1-positive aggregates in NSC34-hSOD1G93A cells. **(D)** Quantification of neurite length from multiple fields of view was analyzed by the Sholl analysis. **(E**,**F)** Bar graphs showing quantification of cFOS and hSOD1 fluorescence intensities from multiple fields of view. *P < 0.05, a pairwise comparison marked by a horizontal line. Data represent the mean ± SEM, statistical significance was assessed by one-way ANOVA followed by LSD-*t* test. **(G,H)** Similar ET-A and ET-B expression are observed in the three cell groups. Bar = 50 μm in **(A,B,G,H)**. Bar = 20 μm in **(C)**.

Second, we examined the expression of MAP1B, which had been proved to influence the organization and dynamics of microtubules in growing and regenerating axons and growth cones (Vaz et al., 2015; Bora et al., 2021; Strohm et al., 2022), to evaluate the ability of differentiation of different cell lines. Even though the three cell groups extended neurites after 24 h, this characteristic was damaged in NSC34-hSODG93A cells. NSC34-E and NSC34-hSOD1WT cells revealed evident increase of neurites upon differentiation, while the neurites of NSC34-hSOD1G93A cells were not obvious (Figure 4A). By the concentric circle (Sholl’s) analysis (Yang et al., 2016), we found that the neurite arborization of NSC34-E and NSC34-hSOD1WT cells had no significant changes while NSC34-hSODG93A cells were poorly differentiated (Figure 4D). The above results demonstrated that the constitutive expression of hSOD1G93A damaged the differentiate ability of NSC34 cells. Meanwhile, it was reported that cFOS can be activated after different stimuli in the CNS. For example, seizure induction caused expression changes of cFOS in the brain (Yang et al., 2019). Simultaneously, cFOS and c-jun take effect in the pathogenesis of AD, which might reflect the initiation of a cell death program in some neurons (Herrera and Robertson, 1996). Therefore, we wonder whether transfection of mutant SOD1 cause expression change of cFOS. Indeed, a remarkably high expression of cFOS was observed in NSC34-hSODG93A cells suggesting that mutant hSOD1G93A may be a kind of stimuli to cells (Figures 4B,E). We further investigated the expression of GFP-hSOD1 *via* immunofluorescence. Different from the uniform hSOD1 distribution in NSC34-hSOD1WT cells, dots indicating protein aggregates was observed in NSC34-hSOD1G93A cells (Figures 4C,F). In addition, consistent with that observed *in vivo*, double-labeling revealed that ET-A and ET-B receptors were both expressed in NSC34 cells (anti-SMI-32 labeled), but no significant expression difference of the two receptors was found among the three cell groups (Figures 4G,H).

### Endothelin-1 is toxic for NSC34-hSOD1G93A cells in a time and concentration dependent manner and endothelin A as well as endothelin B receptors are both implicated in NSC34-hSOD1G93A cell injury

**FIGURE 5:**
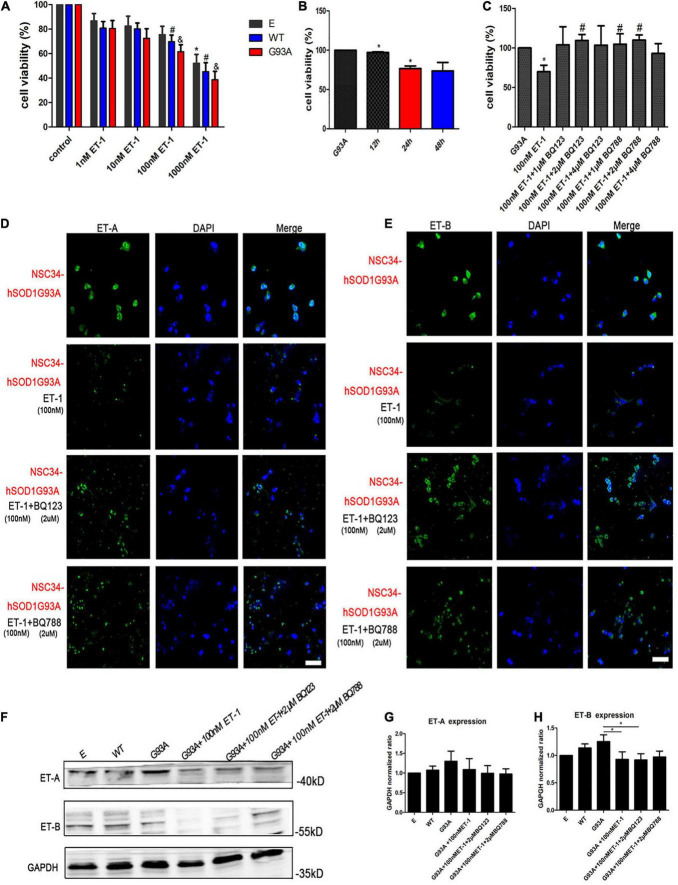
ET-1 decreased NSC34-hSOD1G93A cells viability and ET receptors were responsible for ET-1 toxicity. **(A)** Cell viability of NSC34-E, NSC34-hSOD1WT and NSC34-hSOD1G93A cells treated with ET-1 (1, 10, 100 or 1,000 nM) for 24 h. *P < 0.05, *vs*. NSC34-E cells in control; ^#^*P* < 0.05, *vs*. NSC34-hSOD1WT cells in control; ^&^*P* < 0.05, *vs*. NSC34-hSOD1G93A cells in control. **(B)** Cell viability of NSC34-hSOD1G93A cells treated with 100 nM ET-1 for 12, 24, or 48 h. *P < 0.05, *vs*. NSC34-hSOD1G93A cells. **(C)** Cell viability of NSC34-hSOD1G93A cells after 24 h treatment with ET-1 (100 nM) in the presence of BQ123 (1 μM, 2 μM, 4 μM) or BQ788 (1 μM, 2 μM, 4 μM). *P < 0.05, *vs*. NSC34-hSOD1G93A cells; ^#^*P* < 0.05, *vs*. NSC34-hSOD1G93A cells with 100 nM ET-1 treatment. **(D,E)** Confocal images of ET-A and ET-B expression after 24 h intervention of ET-1 (100 nM), ET-1 + BQ123 (2 μM), or ET-1 + BQ788 (2 μM). Bar = 50 μm. **(F–H)** Western blot analysis showing the expression of ET receptors in three cell groups and ET-1 effect on ET-A and ET-B expression. *P < 0.05, a pairwise comparison marked by a horizontal line. Data represent the mean ± SEM of at least three independent experiments, statistical significance was assessed by one-way ANOVA followed by LSD-t test and Tamhane’s T2 test.

ET-1 in the brain is believed to be deeply involved in the central autonomous control and the subsequent cardiac respiratory homeostasis. It may play a role as a neuromodulator or hormone in the way of autocrine/paracrine locally, or be widely transmitted through cerebrospinal fluid (CSF). The concentration of ET-1 in CSF is higher than that in plasma. Through literature review, we have learned about the physiological, the pathological range of ET-1 in the brain, or the comparison of both (Tu et al., 2015). In a previous study, ET-1 exerted a toxic effect on the motor neuron cultures in a time- and concentration-dependent manner, with an exposure to 100–200 nM ET-1 for 48 h resulting in 40–50% cell death, which can indirectly illustrate the sensitive concentration range of neurons for ET-1 (Ranno et al., 2014). To further examine the effect of ET-1 on MNs survival in our experiment, cells were treated for different lengths of time and with different concentrations of the peptide. An exposure to 1, 10, 100, 1,000 nM ET-1 for 24 h revealed a concentration-dependent toxic effect on the three cell groups (Figure 5A). The CCK-8 results showed that the viability of NSC34-hSOD1G93A cells as well as NSC34-hSOD1WT cells decreased as the ET-1concentration increased, and the same ET-1 intervention caused a slight but no statistically significant decrease of cells viability in NSC34-E group. The relatively low concentrations (100 nM) which had been used frequently (Ranno et al., 2014) to study the toxic effects of ET-1 was also approximate near the median inhibitory concentration (IC50) in our experiment, therefore, 100 nM was chosen for further experiments. Furthermore, an exposure to 100 nM ET-1 for 12, 24, and 48 h revealed a time-dependent toxic effect to MNs reaching a plateau at 24 h (Figure 5B). To validate the involvement of ET-A and ET-B in the process of MNs death, the ET-A competitive receptor antagonist BQ123 and ET-B competitive receptor antagonist BQ788 were used. A treatment of BQ123 or BQ788 together with ET-1 rescued the decrease of cell viability caused by ET-1 (Figure 5C) suggesting the involvement of ET-A and ET-B receptors. The ET-1 effect was obviously reversed by BQ123 (2 μM) and BQ788 (2 μM). Then, we asked whether ET-1 influenced the expression of ET-Rs on the cell model. Double-labeling revealed that ET-1 impaired ET-A and ET-B expression in NSC34-hSOD1G93A cells. When BQ123 or BQ788 was added, ET-Rs expression was greatly preserved (Figures 5D,E). For further quantification, western blot analysis was performed. No significant difference of ET-A or ET-B expression was observed among NSC34-E, NSC34-hSOD1WT and NSC34-hSOD1G93A cells. ET-1 treatment on NSC34-hSOD1G93A cells caused significant decrease of ET-B expression while no significant effect was reached on ET-A expression. In addition, BQ788 rescued the ET-B reduction caused by ET-1 treatment (Figures 5F–H).

The present results suggested that pathologically increased ET-1 might have a potential risk of inducing neuronal death and *via* using pharmacological approaches could potentially reverse the harmful effect. However, caution needs to be paid to further investigate the changes of downstream signaling pathways and function-specificity behind ET-1/ET-Rs signaling axis.

### Quantitative proteomic analysis among NSC34-hSOD1WT, NSC34-hSOD1G93A, and NSC34-hSOD1G93A cells treated with endothelin-1

**FIGURE 6:**
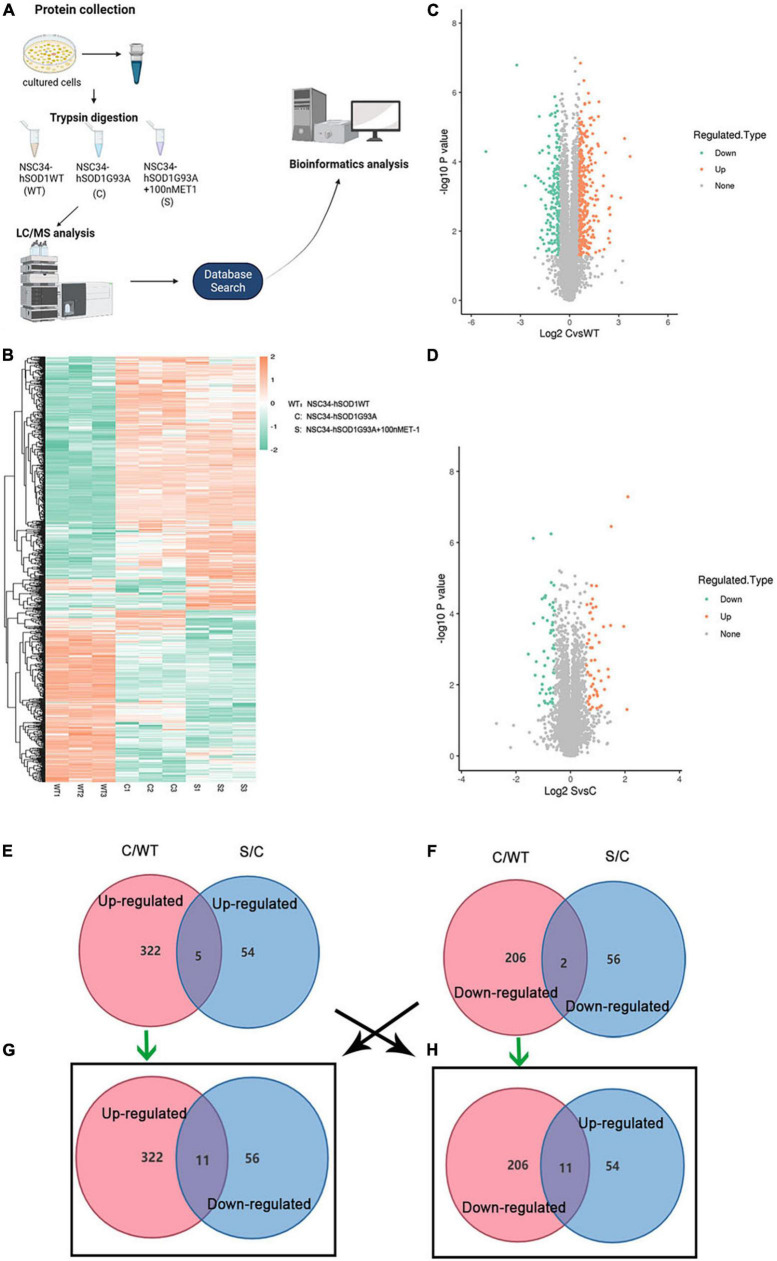
Differentially expressed proteins (DEPs) were evaluated by label-free proteomics. **(A)** Schematic diagram of experimental flow for quantitative proteomic analysis based on the ALS cells models. For each treatment, three biological replicates were prepared. **(B)** Heatmap showing DEPs in WT, C and S cells. **(C,D)** Volcano plots comparing protein abundance in WT, C and S cells. Each dot represents a single quantified protein. Proteins differentially expressed with fold change over 1.5 and *P* < 0.05 were marked in color (*n* = 3 independent biological samples). **(E–H)** Venn diagrams of enriched proteins identified in both C/WT and S/C groups. WT, NSC34-hSOD1WT cells. C, NSC34-hSOD1G93A cells. S, NSC34-hSOD1G93A cells treated with 100 nM ET-1. Pink represents the C/WT comparison group and blue represents the S/C comparison group.

**FIGURE 7:**
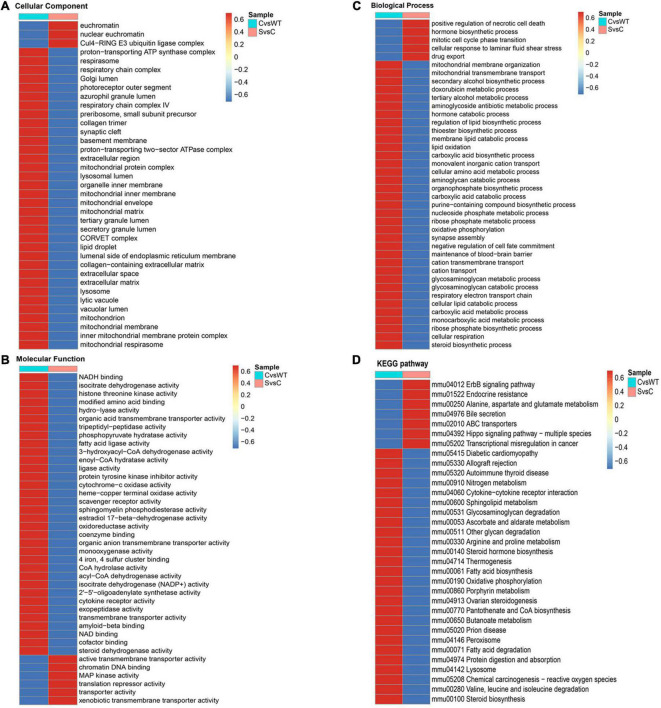
GO enrichment cluster analysis and KEGG pathways cluster analysis of differentially expressed proteins (DEPs) between C/WT and S/C group. **(A)** GO enrichment cluster analysis of DEPs based on cell component. **(B)** GO enrichment cluster analysis of DEPs based on molecular function. **(C)** GO enrichment cluster analysis of DEPs based on biological process. **(D)** KEGG pathway enrichment cluster of DEPs. WT, NSC34-hSOD1WT cells. C, NSC34-hSOD1G93A cells. S, NSC34-hSOD1G93A cells treated with 100 nM ET-1.

To investigate the underlying pathogenesis of ET-1/ET-Rs signaling axis, we performed label-free LC-MS/MS proteomics analysis on NSC34-hSOD1WT (WT) cells, NSC34-hSOD1G93A (C) cells and NSC34-hSOD1G93A treated with 100 nM ET-1 (S) cells (Figure 6A). The above three groups were analyzed, and heatmap graphs of the hierarchical cluster were constructed to visualize the overall broad modulations of proteomes (Figure 6B). For confirmation of differentially expressed proteins (DEPs), the cutoffs for the fold change of abundance and *P*-value were set to 1.5 and 0.05, respectively. In this experiment, compared with WT cells, there were 322 up-regulated and 206 down-regulated DEPs in C cells (Figure 6C). 110 proteins (54 up-regulated proteins and 56 down-regulated proteins) were significantly modulated in S cells vs. C cells (Figure 6D). Interestingly, between the two comparable C/WT and S/C groups, 5 common up-regulated proteins (Figure 6E) and 2 common down-regulated proteins (Figure 6F) were identified. However, when the opposite tendency was analyzed, 11 common DEPs were found between the two comparable C/WT and S/C groups (Figures 6G,H). A total of 29 common proteins were identified in the C/WT and S/C groups and listed in the table (Supplementary Table 1). Subsequently, we found that some of the common proteins-ATP synthase protein 8 (Mtatp8) (Supplementary Figure 3), BCL2/adenovirus E1B 19 kDa protein-interacting protein 3-like (Bnip3l) (Figure 7D), ATP synthase-coupling factor 6, mitochondrial (Atp5pf) (Figure 7D), Valine-tRNA ligase, mitochondrial (Vars2) (Supplementary Figure 3) and Protein KIBRA (Wwc1) (Figure 7D) were strongly enriched in reactive oxygen species (Figure 7D), oxidative phosphorylation (Supplementary Figure 3), mitophagy-animal (Figure 7D), Alzheimer disease (Figure 7D), Parkinson disease (Figure 7D), Huntington disease (Figure 7D), Prion disease (Figure 7D), aminoacyl-tRNA biosynthesis (Supplementary Figure 3), ABC transporters (Figure 7D), Hippo signaling pathway (Figure 7D) and so on through Kyoto Encyclopedia of Genes and Genomes (KEGG) pathway analysis, which revealed that ET-1 treatment might play a role in these signaling pathways.

To investigate the associated functions of the identified DEPs in the cells model based on ALS disease, we obtained distinctive KEGG pathway and Gene Ontology (GO) terms analysis between the two comparable groups of C/WT and S/WT (Supplementary Figure 3 and Figure 7). The KEGG pathway is used for analyzing the correlation among known molecular functions such as metabolic pathways, formation of complexes, and biochemical reactions (Wang et al., 2019). As it may be seen in Supplementary Figure 3 of C/WT group, KEGG pathway analysis suggested that the up- and down-regulated DEPs were enriched in oxidative phosphorylation, reactive oxygen species, various neurodegenerative disease such as Parkinson disease and Alzheimer disease. For proteins that enriched in the S/C group, the KEGG analysis showed a strong interaction with a series of pathways related with neurodegenerative disease, such as Hippo signaling pathway-multiple species (Gogia et al., 2019), ABC transporters (Pahnke et al., 2021), oxidative phosphorylation, and ErbB signaling pathway (Supplementary Figure 3; Sun et al., 2020). In summary, these results suggest that ET-1 could induce a series of events in the cells model. In the part of GO enrichment analysis, C/WT group was highly enriched in extracellular region and synaptic cleft based on cellular component while S/C group was related to Gul4-RING E3 ubiquitin ligase complex and nuclear euchromatin (Figure 7A). In C/WT comparable group, significant changes occurred in some molecular functions like protein tyrosine kinase inhibitor activity, sphingomyelin phosphodiesterase activity while more DEPs were associated with xenobiotic transmembrane transporter activity, chromatin DNA binding and MAP kinase activity in the S/C group (Figure 7B). According to biological process enrichment, in C/WT group, mitochondrial membrane organization, mitochondrial transmembrane transport and organophosphate biosynthetic process were greatly enriched, while cellular response to laminar fluid shear stress, mitotic cell cycle phase transition and drug export were significantly involved in the S/C group (Figure 7C). KEGG pathway analysis showed that DEPs are located in important pathways such as allograft rejection and prion disease in C/WT group, however, in S/C group, DEPs were specifically enriched in Hippo signaling pathway-multiple species, ErbB signaling pathway and so on (Figure 7D).

### Proteomic analysis revealed endothelin-1 affects neuron survival through the functions of differentially expressed proteins

**FIGURE 8:**
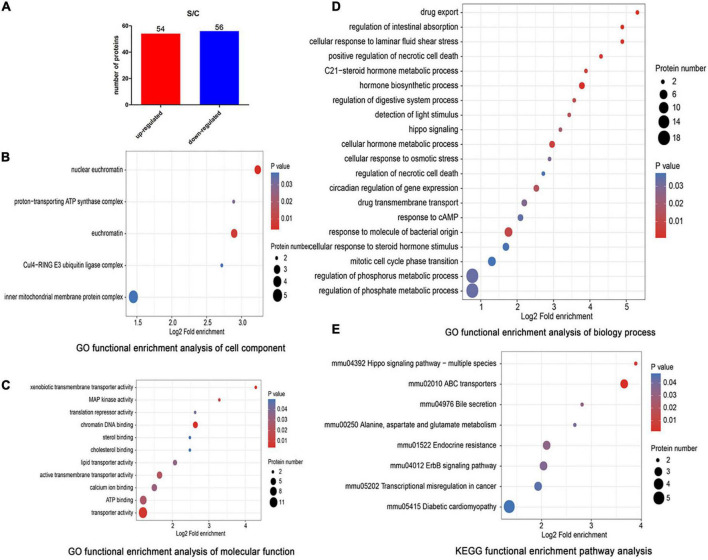
Proteomic analysis of the S/C comparable group. **(A)** In the S/C comparable group, we identified 110 DEPs, including 54 up-regulated and 56 down-regulated genes (using a 1.5-fold cutoff, *P* < 0.05). **(B)** The top 20 enriched GO terms of DEPs (fold change > 1.5) based on cellular component. **(C)** The top 20 enriched GO terms of DEPs (fold change > 1.5) based on molecular functions. **(D)** GO enrichment analysis of DEPs based on biological process (fold change > 1.5). **(E)** KEGG enrichment analysis following S/C group (fold change > 1.5). C, NSC34-hSOD1G93A cells. S, NSC34-hSOD1G93A cells treated with 100 nM ET-1.

The results of proteomic analysis exhibited that compared with C cells group (as positive control), there were 54 upregulated and 56 downregulated proteins after ET-1 intervention (Figure 8A). Gene ontology (GO) functional classification analysis showed that the DEPs in S/C comparable group mainly fell into cellular process, biological regulation, metabolic process, response to stimulus, multicellular organismal process, developmental process, localization and other biological process (Supplementary Figure 4). The main results of the GO enrichment analysis are shown in Figures 8B–E. In particular, the DEPs based on cell components were enriched mainly in nuclear euchromatin and proton-transporting ATP synthase complex (Figure 8B). Molecular functions were enriched mainly in terms related to xenobiotic transmembrane transporter activity, MAP kinase activity, translation repressor activity and chromatin DNA binding (Figure 8C). Then, the DEPs were enriched in drug export, regulation of intestinal absorption, cellular response to laminar fluid shear stress, positive regulation of necrotic cell death, C2-steroid hormone metabolic process, hormone biosynthetic process, hippo signaling and other biological process (Figure 8D). Meanwhile, KEGG analysis showed that the DEPs were primarily enriched in Hippo signaling pathway-multiple species and ABC transporters (Figure 8E). By combining the results, we found that ET-1 significantly modulated protein metabolic process, signal transduction and response to stimulus. The subcellular localization analysis showed that most of the DEPs were distributed in the nucleus (27.46%, 30 proteins), cytoplasm (23.3%, 27 proteins), mitochondria (17.42%, 19 proteins), extracellular (12.88%, 14 proteins), and plasma membrane (10.98%, 12 proteins). The data revealed that except for the nucleus, the largest proportion of DEPs was located in cytoplasm, followed by mitochondria and extracellular (Supplementary Figure 4).

### Mass spectrometry analysis of parallel reaction monitoring validation candidate peptides

To further confirm the cells proteome results related with ET-1 treatment, we performed PRM assay rather than the traditional western blot, which selected specific peptides or target peptides and then made the target protein quantified (Xu et al., 2018). According to risk assessment and limited to characteristics and abundances of 110 DEPs in the S/C comparable group, 16 target proteins were evaluated and then quantified in the experiment. At present, a total of 16 DEPs which had not previously been clearly reported to be relevant with ET-1 intervention on NSC34-hSOD1G93A cells was verified, including 9 up-regulated proteins and 7 down-regulated proteins identified in the basis of label-free quantitative analysis. The fragment ion peak of one peptide and corresponding proteins were displayed in Supplementary Table 2. 16 of identified proteins presented the same changing trend and the former 13 proteins that were identified significantly altered, demonstrating the consistency between PRM and LC-MS/MS proteome (Supplementary Table 3). These results suggested that the related effects of ET-1 on the NSC34-hSOD1G93A cells may closely connected with the functions of these proteins. Thus, ET-1 might influence kinds of signaling pathways to impose MNs damaging effects.

**FIGURE 9:**
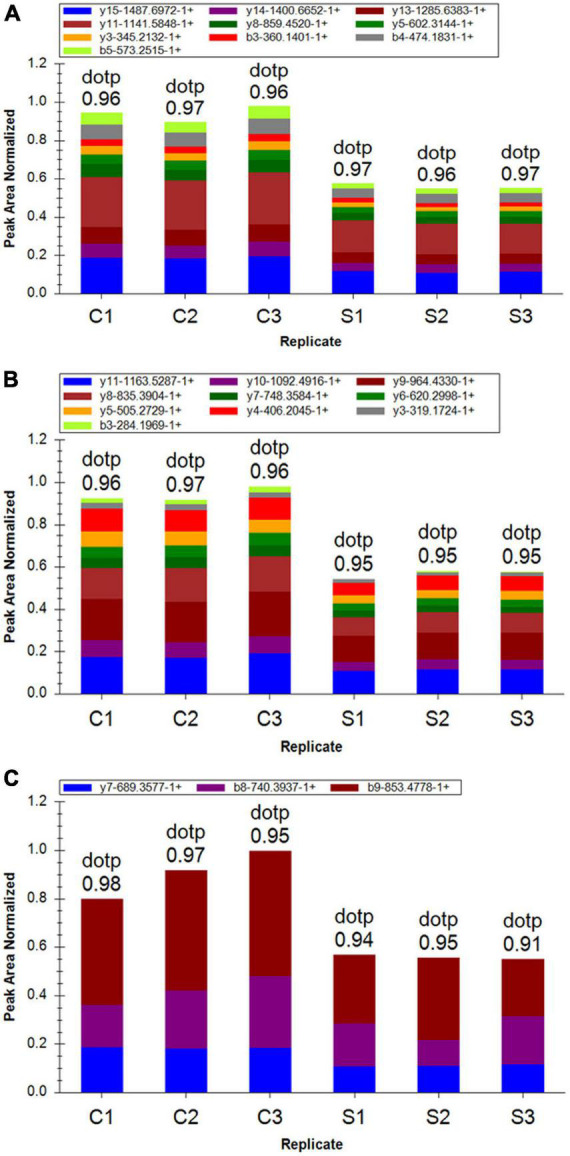
Differentially expressed proteins (DEPs) quantification by mass spectrometry-based targeted proteomics-parallel reaction monitoring (PRM). **(A)** Fragment ion peak area distribution of TEENVSDGSPNAGTVEQTPK peptide for Cdkn1b (P27). **(B)** Fragment ion peak area distribution of VLAQESQDVSGSR peptide for Cdkn1b (P27). **(C)** Verification of peptide VALGDGVQLPPGDYSTTPGGTLFSTTPGGTR targeting protein Eif4ebp1.

**FIGURE 10:**
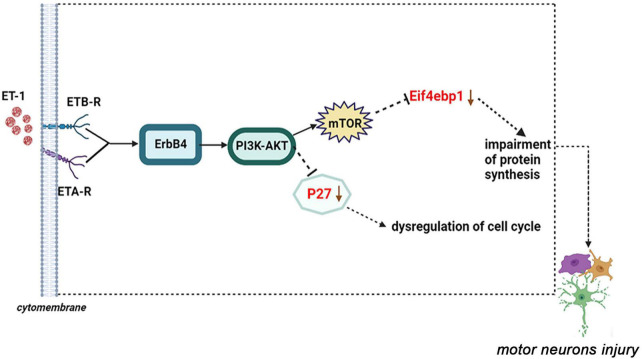
A proposed model of ET-1/ET-Rs axis interacts with the downstream molecular-P27 or Eif4ebp1 and the dysfunction of these genes may result in motor neurons injury (ErbB signaling pathway: pathway ID# mmu04012).

Among these verified differential proteins, we noticed that Cdkn1b (Figures 9A,B) and Eif4ebp1 (Figure 9C), significantly down-regulated when treated with ET-1, were particularly enriched in ErbB signaling pathway (pathway ID# mmu04012, Supplementary Figure 5). ErbB pathway dysregulation has been proposed to play a pathogenic role in ALS, especially ErbB4, a member of the epidermal growth factor (EGF) subfamily of receptor tyrosine kinases (RTKs) (Casanovas et al., 2017). These results suggest that the effects of ET-1 on NSC34-hSOD1G93A cells are closely concerned with the functions of mentioned proteins above and ET-1 can exert the related effects mainly through cell cycle and protein synthesis regulation. These proteins are anticipating targets for the study of ALS pathogenesis (Figure 10).

## Discussion

The loss of upper and lower motor neurons in the structures such as cortex, brain stem and spinal cord, is a major contributor to the pathogenic mechanism of ALS, while neither highly effective treatments nor potent protective approaches have yet been identified (Yamanaka and Komine, 2018). In this study, we focused on exploring the location and expression of ET-1/ET-Rs on ALS mice then defining the effects of ET-1 intervention on NSC34-hSOD1G93A cells, and attempted to elucidate the mechanism of MNs injury through proteomics analysis. In general, our results showed ET-1 was mainly up-regulated in astrocytes, especially at the end stage of SOD1-G93A mice, which was in consistence with the previous study (Ranno et al., 2014). However, ET-A and ET-B receptors were mainly located in neurons and ET receptors-positive neurons were greatly reduced in end stage SOD1-G93A mice in spite of the expression of two receptors found in some kind of non-neuron cells occasionally. This provided a theoretical foundation for our following *in vitro* study on defining the effects of ET-1/ET-Rs on MNs and the expression profiles of critical proteins mediated by the exposure of ET-1.

Both ET-A and ET-B receptors were expressed on the motoneuron-like cells model, which is consistent with our *in vivo* cellular localization of ET-Rs on ALS mice model. ET-1 induced MNs death *in vitro* and the toxic effect could be partially blocked by specific antagonists BQ123 and BQ788, revealing an involvement of ET-A and ET-B receptors. Interestingly, we observed that ET-1 intervention decreased the expression of ET-A and ET-B receptors and reduced the cell viability of NSC34-hSOD1G93A cells. From these, we speculate that the increase of ET-1 expression in the spinal cords of SOD1-G93A mice may be *per se* toxic for MNs. Meanwhile, ET-1 could be an important indicator of changes in response to certain diseases and a potential candidate as a therapeutic target (Koyama et al., 2019). In this study, proteomics approach (Monti et al., 2019) was used to understand the possible molecular mechanism of ET-1 induced MNs damage in the *in vitro* ALS model. Proteins highly enriched in the S/C comparable group play a significant role in pathways such as Hippo signaling pathway-multiple species, ATP-binding cassette (ABC) transporters. According to previous studies, Hippo signaling pathways seem to be interesting therapeutic targets, which can alleviate the onset or progression of neurodegenerative diseases like ALS (Gogia et al., 2019). Hippo signaling dysregulation has also been identified in models of Alzheimer’s disease and neuronal stem cells (Wu et al., 2003; Gogia et al., 2021; Xu et al., 2021). Thus, ET-1/ET-Rs/Hippo can be an interesting link in the neurodegenerative disorder like ALS. For another, one mechanism causing difficulties in remedying CNS diseases using pharmacotherapy (e.g., epilepsy and HIV-related neurological conditions) is the obstacle provided by the activity of ABC transporters in brain barrier tissues (Eng et al., 2022). The transporters are ATP-driven efflux pumps with remarkably broad substrate specificity and are answerable for the high urine-to-plasma and bile-to-plasma concentration ratios seen for some xenobiotics (excretory function) and inhibit many xenobiotics to enter the CNS (barrier function) (Hagos et al., 2019). In light of the DEPs enriched in ABC transporters in the proteomic results of *in vitro* ALS model, understanding the alterations in ABC transporters induced by ET-1 intervention becomes increasingly relevant.

In order to validate the proteomic data, we selected several DEPs for more accurate quantitative evaluation by PRM. We found that 13 of these DEPs showed the significantly same expression tendency as that inferred through Label-free proteomics analysis. Strikingly, our results revealed that among these verified proteins, the protein expression of Eif4ebp1 and P27 was significantly down-regulated after ET-1 treatment on NSC34-hSOD1G93A cells and they were particularly enriched in ErbB signaling pathway through KEGG enrichment analysis (pathway ID# mmu04012). ErbB pathway dysregulation has been proposed to play a pathogenic role in ALS, especially one of the subtypes-ErbB4 (Sun et al., 2020). ErbB4 forms a homodimer or a heterodimer with ErbB2 or ErbB3 and is activated upon binding of neuregulins (NRGs) to the extracellular ligand-binding domain of ErbB4 (D’Antoni et al., 2017). ErbB4 could also mainly contribute to downstream signaling pathways phosphoinositide 3-kinase (PI3K)/Akt serine/threonine kinase (Akt) pathway activation (pathway ID# mmu04012), which had been tested for the possible involvement of its toxic action in ALS (Codeluppi et al., 2009). Studies suggested the existence of a causal relationship between PI3K/Akt pathway and mechanistic target of rapamycin kinase (mTOR) signaling in neurodegeneration and CNS injury. For instance, in a model of spinal cord injury, there was mTOR activation by Akt (Granatiero et al., 2021). Further, members of the PI3K family are lipid kinases participated in multiple cellular processes, such as proliferation, differentiation, migration, metabolism and survival (Li et al., 2022). Subsequently, PI3K will activate the downstream Akt to inner membranes and phosphorylates Akt on its serine/threonine kinase sites (Thr308 and Ser473). Recruited Akt participates in the downstream mTORC1 mediated response to biogenesis of protein and ribosome (Fan et al., 2018). Moreover, mTOR activation leads to the phosphorylation of Eif4ebp1/4EBP1 at Thr37/46 (Morita et al., 2015), a pivotal regulator of protein translation and cell growth (Fan et al., 2018), which simultaneously could be seen as its implication in transducing survival signals based on our proteomic results. Importantly, our results also showed PI3K/Akt signaling pathways activated cyclin-dependent kinase inhibitor p27^kip1^ (Cdkn1b/P27) after ET-1 treatment on NSC34-hSOD1G93A cells, a critical determinant target for cell cycle progression, which was a vital regulation target of mitogenic signals (Muñoz et al., 2008). The alterations in P27 signaling molecules had been detectable in lymphoblasts from Alzheimer’s disease (AD) patients, a kind of neurodegenerative disorders (Bartolomé et al., 2009).

From what has been discussed above, we clarify the importance of Cdkn1b (P27) and Eif4ebp1, as biomarkers for more comprehensively understanding and identifying the pathogenesis of ALS responding to ET-1 intervention. Thus, we drew a diagram of ET-1/ET-Rs axis inducing MNs damage through the ErbB4-PI3K/Akt-mTOR-Eif4ebp1 or ErbB4-PI3K/Akt-P27 pathway (Figure 10). By combining our proteomic data with our preliminary observational data, the latter bioinformatics analysis provides us with a more comprehensive understanding of the mechanism of ET-1 intervention on ALS *in vitro* model, which no studies so far have addressed. In the future study, more attention should be paid to identify regulatory mechanisms of ErbB4-PI3K/Akt-mTOR-Eif4ebp1 or ErbB4-PI3K/Akt-P27 pathways and the potential role of ET-1 in the treatment of ALS.

There are several limitations of this study, requiring further examination and additional research. First, NSC34-hSOD1G93A cells model treated with ET-1 do not represent real *in vivo* ALS models. Further in-depth analysis of the ET-1 intervention effects *in vivo* models of ALS will be indispensable to prove preclinical efficacy by attenuating ET-1 induced neuronal toxicity. Second, the exact molecular pathways conducting the effect of ET-1/ET-Rs needs to be further verified.

## Conclusion

ET-1/ET-Rs pathway plays a role in the pathogenesis of ALS. ET-1 induction of activated astrocytes may be toxic to ET-R-expressing motor neurons and multiple molecular mechanisms contribute to this damaging effect. Targeting ET-1 could be a potential therapeutic strategy for ALS.

## Data availability statement

The datasets presented in this study can be found in online repositories. The names of the repository/repositories and accession number(s) can be found below: ProteomeXchange: PXD037555.

## Ethics statement

The animal study was reviewed and approved by the Ethics Committee of the Second Hospital of Hebei Medical University.

## Author contributions

YG designed the study. YZ performed the research, analyzed the data, and wrote the manuscript. LC, ZL, and YW assisted some of the experiments. DL analyzed the data and revised the manuscript. YG and YZ revised the manuscript. All authors have read and approved the final version of the manuscript.
