# Supplementary material for: Endothelin-1, over-expressed in SOD1G93A mice, aggravates injury of NSC34-hSOD1G93A cells through complicated molecular mechanism revealed by quantitative proteomics analysis
Source: Front Cell Neurosci. 2022 Dec 1;16:1069617. doi: 10.3389/fncel.2022.1069617 (PMC9752095; doi:10.3389/fncel.2022.1069617)
Supplement: Supplementary file 1 [file Data_Sheet_1.DOCX]

**Supplementary Methods**

**Sample Preparation**

After resuscitation, the cells were cultured for 3-5 generations, and the follow-up experiments were carried out when the cells were in the best condition. The experimental group and the control group cells were all passaged from the same culture dish. Exposure to ET-1 for 24 h, the cells were collected, washed with pre-cooled PBS for 3 times, centrifuged at 5000 rpm for 5 min at 4 °C to pellet the cells and discard the supernatant, then put the samples in liquid nitrogen immediately and stored at -80 °C for later use. Next, samples were taken out and sonicated for three times on ice using a high intensity ultrasonic processor (Scientz) in the lysis buffer (8 M urea, 1% protease inhibitor cocktail). After centrifugation at 12,000 g at 4 °C for 10 min, the remaining debris was removed. In the end, the supernatant was harvested and the protein concentration was calculated with BCA kit in accordance with the manufacturer’s recommendations.

The protein solution was reduced with 5 mM dithiothreitol for 30 min at 56 °C for digestion, followed by alkylating with 11 mM iodoacetamide for 15 min at room temperature in the dark. To reduce urea concentration < 2 M, the protein sample was diluted by adding 100 mM TEAB. Trypsin was added to digest the protein sample overnight firstly at the ratio of 1:50 trypsin-to- protein, and 1:100 trypsin-to-protein mass ratio for the next digestion for 4 h. At last, the peptides were desalted by C18 SPE column.

**Database Search**

Allowing up to 2 missing cleavages, trypsin/P was regarded as cleavage enzyme. For precursor ions, the mass tolerance was set as 10 ppm in first search, 5 ppm in main search, and the mass tolerance was set as 0.02 Da for fragment ions. The carbamidomethylation of cysteines was considered as fixed modifications, acetylation on protein N-terminal and oxidation on Met were regarded as [alterable](javascript:;) modifications. FDR was regulated to < 1%.

**Parallel Reaction Monitoring (PRM) analysis**

Proteins were extracted and digested using trypsin. Then, the tryptic peptides were dissolved in 0.1% formic acid (solvent A), directly loaded onto a home-made reversed-phase analytical column. The gradient was comprised of an increase from 6% to 23% solvent B (0.1% formic acid in 98% acetonitrile) over 38 min, 23% to 35% in 14 min and climbing to 80% in 4 min then holding at 80% for the last 4 min, all at a constant flow rate of 700 nL/min on an EASY-nLC 1000 UPLC system.

The peptides were subjected to NSI source followed by tandem mass spectrometry (MS/MS) in Q ExactiveTM Plus (Thermo) coupled online to the UPLC. The electrospray voltage applied was 2.1 kV. The m/z scan range was 306 to 1032 for full scan, and intact peptides were detected in the Orbitrap at a resolution of 70,000. Peptides were then selected for MS/MS using NCE setting as 27 and the fragments were detected in the Orbitrap at a resolution of 17,500. A data-independent procedure that alternated between one MS scan followed by 20 MS/MS scans. Automatic gain control (AGC) was set at 3E6 for full MS and 1E5 for MS/MS. The maximum IT was set at 50 ms for full MS and auto for MS/MS. The isolation window for MS/MS was set at 1.6 m/z.

The resulting MS data were processed using Skyline (v.21.1). Peptide settings: enzyme was set as Trypsin [KR/P], Max missed cleavage set as 0. The peptide length was set as 7-25, Variable modification was set as Carbamidomethyl on Cys and oxidation on Met, and max variable modifications was set as 3. Transition settings: precursor charges were set as 2, 3, ion charges were set as 1, ion types were set as b, y, p. The product ions were set as from ion 3 to last ion, the ion match tolerance was set as 0.02 Da.

**Supplementary results**

**Supplementary Figure S1**

**
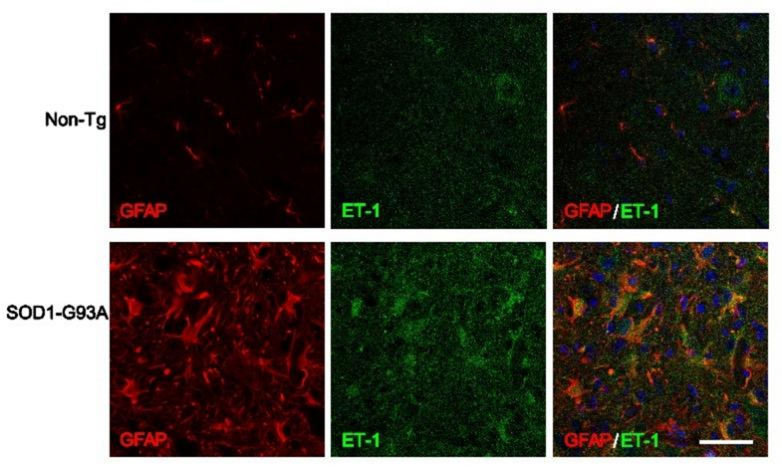
**

**Suppl. Fig. S1 Cellular localization of ET-1 in the lumbar spinal cord of nontransgenic (NTG) mice and SOD1-G93A transgenic mice at end stage.** ET-1 positive cells were expressed mainly in proliferating GFAP-positive astrocytes in lumbar spinal cord of SOD1-G93A mice.

**Supplementary Figure S2**

**
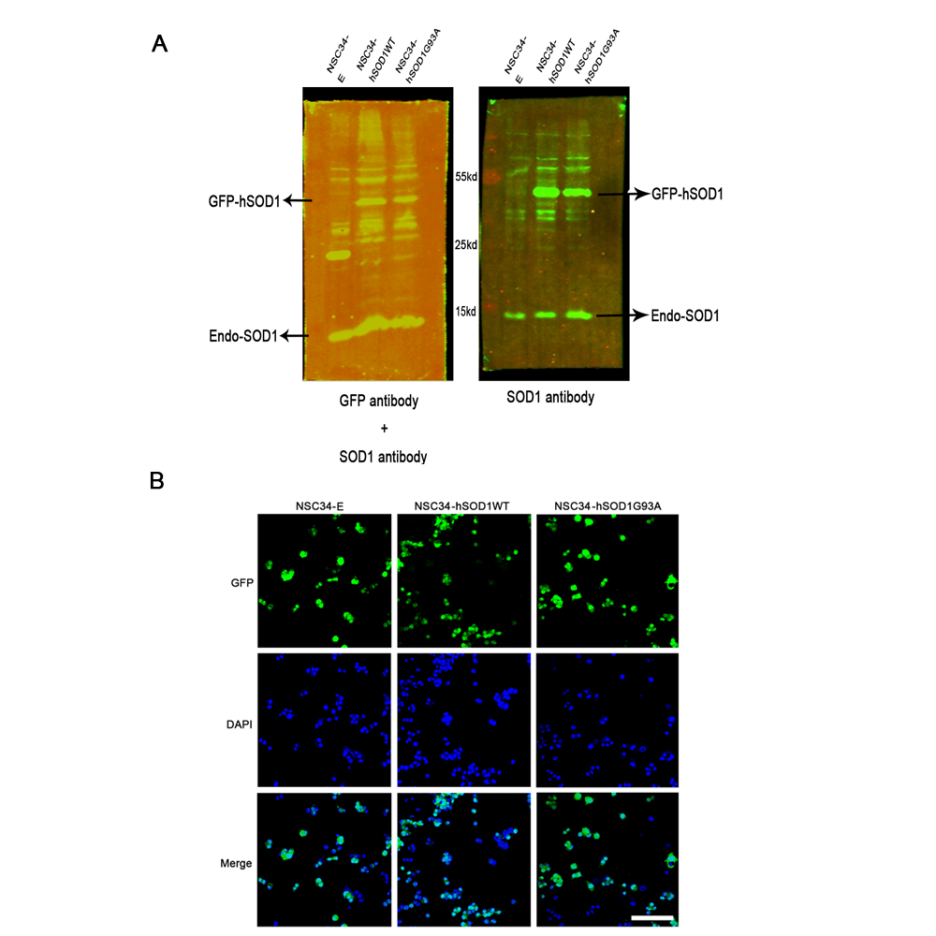
**

**Suppl. Fig. S2 Identification of the motor neuronal cell model of ALS.** (A) Expression of GFP in NSC34-E cells, GFP-hSOD1 fusion proteins in NSC34-hSOD1WT and NSC34-hSOD1G93A cells, and endogenous SOD1 (Endo-SOD1) expressed in three cell groups. (B) NSC34-E, NSC34-hSOD1WT and NSC34-hSOD1G93A cells co-labeled with anti-GFP (green) and DAPI (blue). Bar = 100 μm.

**Supplementary Figure S3**

**
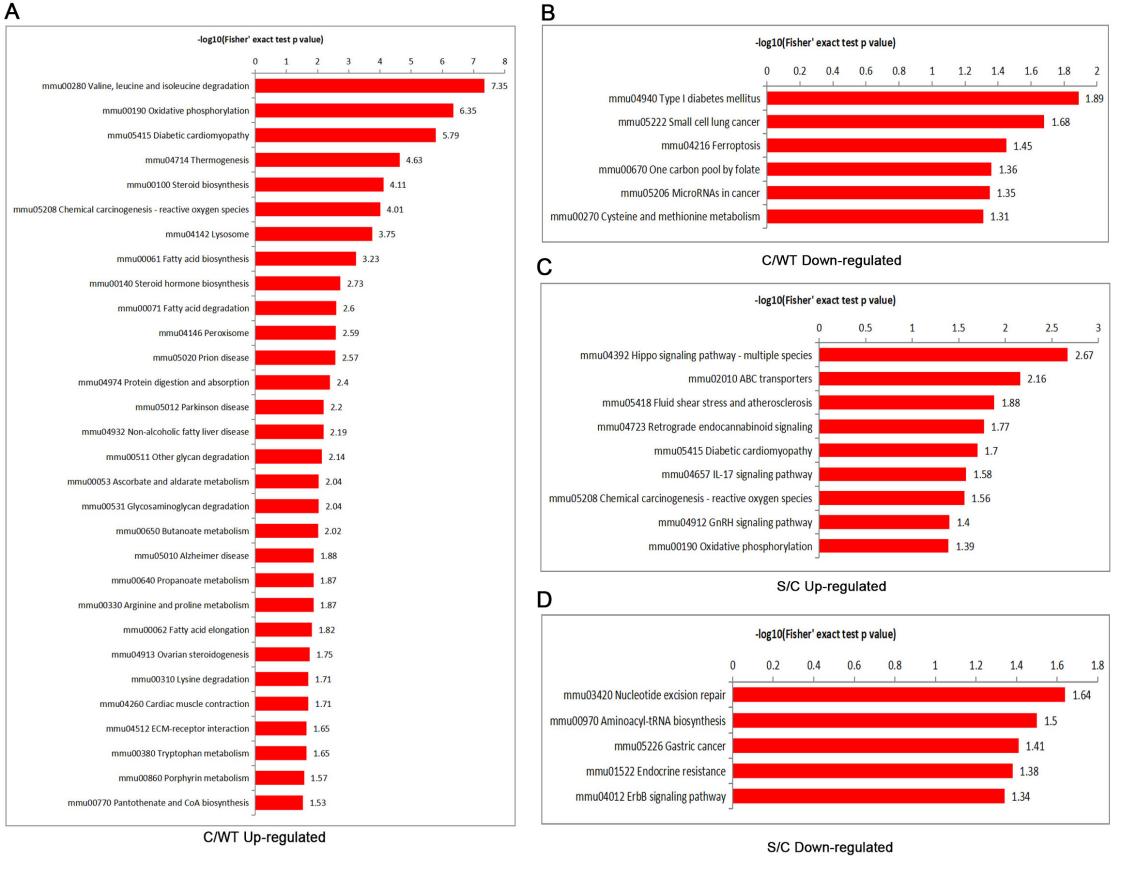
**

**Suppl. Fig. S3 KEGG pathway between C/WT and S/C group.**

(A, B) The enriched KEGG pathway in C/WT group of differentially expressed proteins (fold enrichment> 1.5). (C, D) The enriched KEGG pathway in S/C group of differentially expressed proteins (fold enrichment> 1.5). WT, NSC34-hSOD1WT cells. C, NSC34-hSOD1G93A cells. S, NSC34-hSOD1G93A cells treated with ET-1.

**Supplementary Figure S4**

**
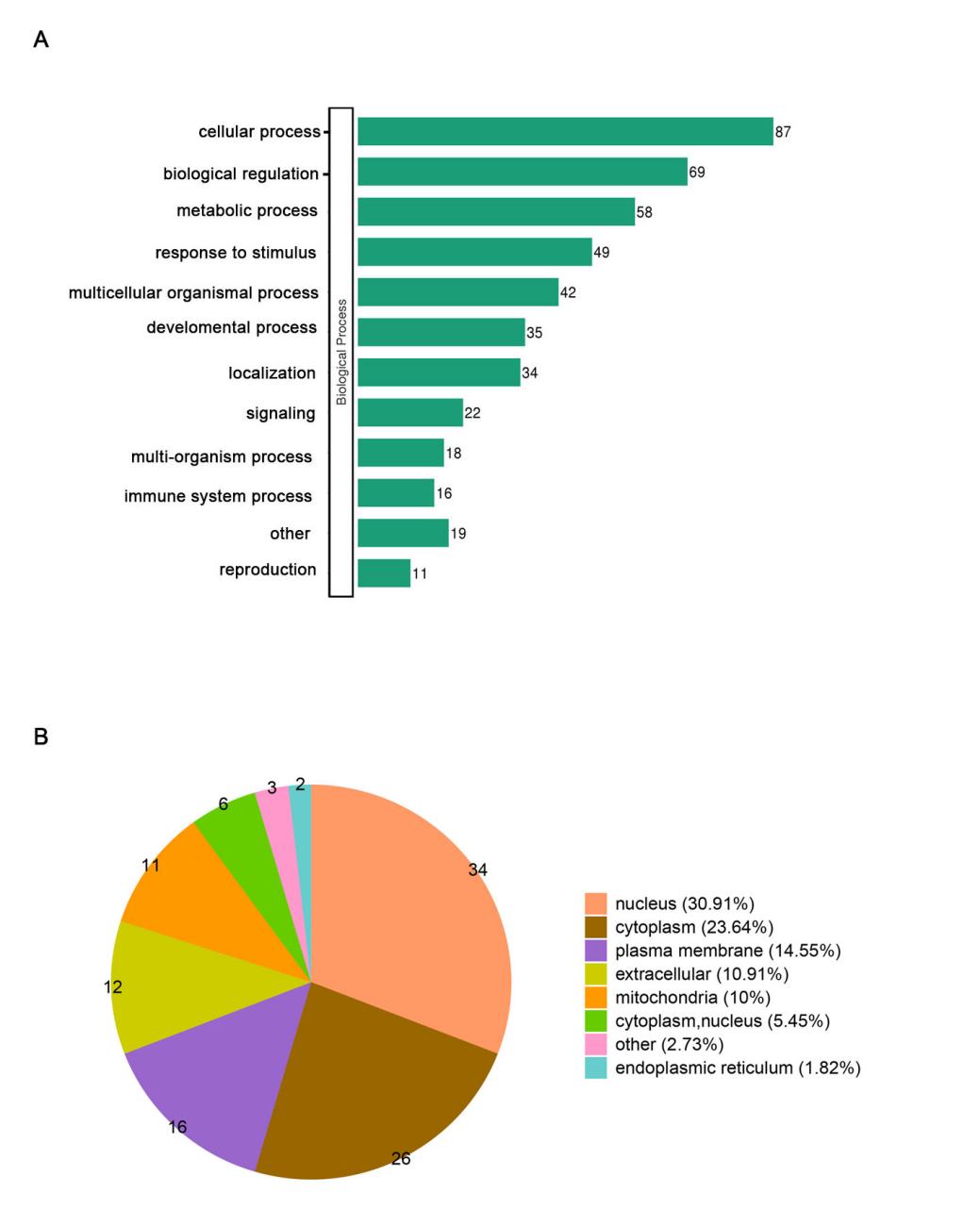
**

**Suppl. Fig. S4 (A)** GO functional classification of DEPs. **(B)** Subcellular localization classification in various subcellular locations.

**Supplementary Figure S5**

**
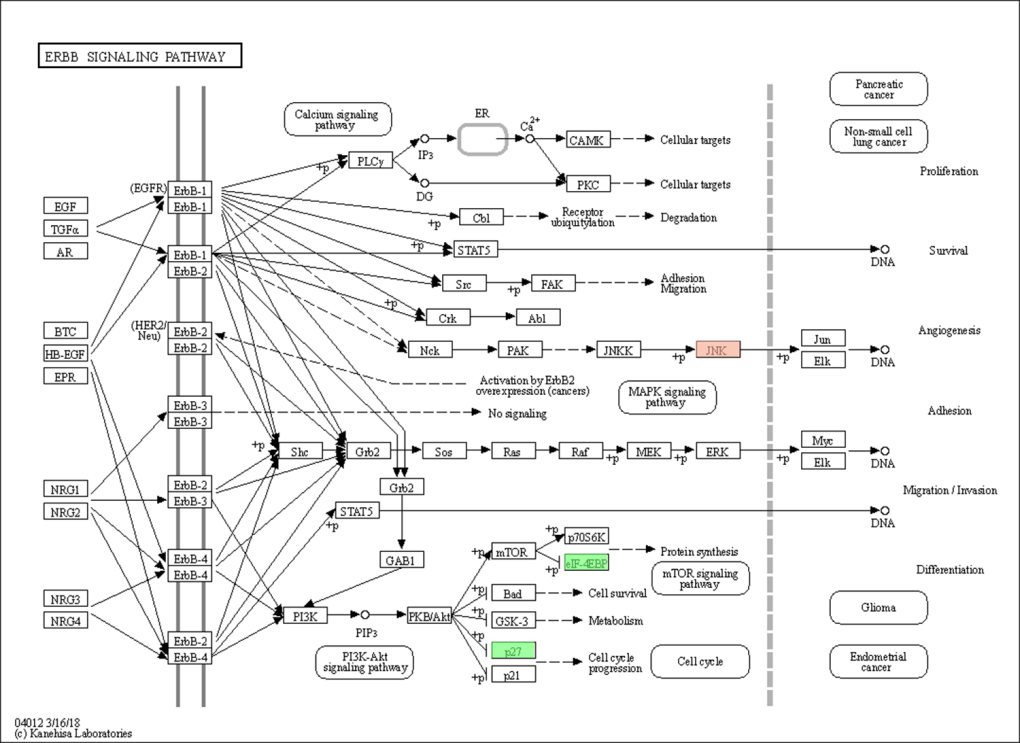
**

**Suppl. Fig. S5** ErbB signaling pathway (ID# mmu04012). The proteins in green background were down-regulated.

**Table S1.** The differentially expressed proteins shared between C/WT and S/C comparable group (fold change>1.5).

| **Protein description** | **Fold change**  **(C/WT)** | **Regulated Type(C/WT)** | **Fold change**  **(S/C)** | **Regulated Type(S/C)** |
| --- | --- | --- | --- | --- |
| Zinc finger MYM-type protein 3 | 2.808 | up | 4.202 | up |
| Zinc finger homeobox protein 3 | 2.677 | up | 1.666 | up |
| Retinoic acid-induced protein 1 | 2.03 | up | 2.375 | up |
| Collagen alpha-1(XV) chain | 1.996 | up | 1.861 | up |
| ATP synthase protein 8 | 1.66 | up | 1.599 | up |
| Jupiter microtubule associated homolog 2 | 0.625 | down | 0.486 | down |
| ATP-dependent translocase ABCB1 | 0.441 | down | 0.563 | down |
| Steryl-sulfatase | 12.874 | up | 0.526 | down |
| Zinc finger protein 37 | 3.449 | up | 0.485 | down |
| Methanethiol oxidase | 3.153 | up | 0.664 | down |
| Prothymosin alpha | 2.508 | up | 0.633 | down |
| Coiled-coil domain-containing protein 127 | 2.417 | up | 0.662 | down |
| Argininosuccinate synthase | 2.297 | up | 1.519 | down |
| Histone H1.0 | 2.135 | up | 0.61 | down |
| Tudor domain-containing protein 7 | 1.979 | up | 0.46 | down |
| BCL2/adenovirus E1B 19 kDa protein-interacting protein 3-like | 1.889 | up | 0.659 | down |
| ATP synthase-coupling factor 6, mitochondrial | 1.684 | up | 0.529 | down |
| Valine--tRNA ligase, mitochondrial | 1.676 | up | 0.637 | down |
| F-box/LRR-repeat protein 8 | 0.655 | down | 1.964 | up |
| RNA-binding protein 3 | 0.648 | down | 1.53 | up |
| Protein SHQ1 homolog | 0.61 | down | 1.698 | up |
| E3 ubiquitin-protein ligase Rnf220 | 0.608 | down | 1.914 | up |
| Nucleolar protein 7 | 0.567 | down | 1.863 | up |
| Vacuolar protein sorting-associated protein 8 homolog | 0.505 | down | 1.954 | up |
| Protein N-terminal asparagine amidohydrolase | 0.455 | down | 2.174 | up |
| Coatomer subunit zeta-2 | 0.442 | down | 2.597 | up |
| Receptor-interacting serine/threonine-protein kinase 3 | 0.397 | down | 1.604 | up |
| Protein KIBRA | 0.36 | down | 2.772 | up |
| Carbonic anhydrase-related protein | 0.298 | down | 1.598 | up |

WT, NSC34-hSOD1WT cells. C, NSC34-hSOD1G93A cells. S, NSC34-hSOD1G93A cells treated with ET-1.

**Table S2.** The fragment ion peak area distribution of identified peptides of differential proteins

| **Protein Accession** | **Peptide** | **Gene**  **Name** | **Retention**  **time** | **S1 Area** | **S2 Area** | **S3 Area** | **C1 Area** | **C2 Area** | **C3**  **Area** |
| --- | --- | --- | --- | --- | --- | --- | --- | --- | --- |
| Q3THK7 | ELDLPEELVSR | Gmps | 45.88 | 3.18e+07 | 3.53e+07 | 3.57e+07 | 3.01e+07 | 2.58e+07 | 3.12e+07 |
| Q3THK7 | VVYIFGPPVK | Gmps | 46.19 | 6.29e+07 | 6.39e+07 | 6.62e+07 | 6.09e+07 | 5.83e+07 | 6.34e+07 |
| P97450 | ELDPVQK | Atp5pf | 12.47 | 2.65e+07 | 3.78e+07 | 3.69e+07 | 5.97e+07 | 7.42e+07 | 4.15e+07 |
| P97450 | QASGGPVDIGPEYQQDLDR | Atp5pf | 33.02 | 3.69e+07 | 3.55e+07 | 3.60e+07 | 6.44e+07 | 6.05e+07 | 5.94e+07 |
| P16460 | APNSPDVLEIEFK | Ass1 | 46.18 | 7.84e+07 | 8.15e+07 | 1.01e+08 | 6.20e+07 | 5.74e+07 | 7.77e+07 |
| P16460 | FAELVYTGFWHSPECEFVR | Ass1 | 52.14 | 1.04e+08 | 8.55e+07 | 1.02e+08 | 5.36e+07 | 6.90e+07 | 5.32e+07 |
| Q8BI72 | NAGDLVPATDETADAESGAR | Cdkn2aip | 24.56 | 2.69e+07 | 2.80e+07 | 2.75e+07 | 2.29e+07 | 2.15e+07 | 1.97e+07 |
| Q8BI72 | GISSSNEGVEEPSK | Cdkn2aip | 11.23 | 1.74e+07 | 1.86e+07 | 1.42e+07 | 1.53e+07 | 1.39e+07 | 1.53e+07 |
| P10922 | VGENADSQIK | H1-0 | 8.79 | 1.42e+07 | 1.54e+07 | 1.66e+07 | 2.65e+07 | 2.75e+07 | 2.64e+07 |
| P10922 | GVGASGSFR | H1-0 | 10.97 | 6.32e+07 | 6.59e+07 | 7.21e+07 | 1.15e+08 | 1.08e+08 | 6.13e+07 |
| O35345 | EPSPPIDEVINTPGVVDR | Kpna6 | 45.88 | 1.80e+07 | 1.89e+07 | 1.85e+07 | 1.33e+07 | 1.38e+07 | 1.61e+07 |
| O35345 | EAAWAITNATSGGTPEQIR | Kpna6 | 36.57 | 1.02e+07 | 1.13e+07 | 1.02e+07 | 1.06e+07 | 8.68e+06 | 8.70e+06 |
| Q9CQY5 | QADEEFQILANSWR | Magt1 | 50.21 | 5.98e+06 | 7.99e+06 | 7.98e+06 | 6.48e+06 | 5.41e+06 | 5.31e+06 |
| Q9CQY5 | GFSAEQIAR | Magt1 | 20.71 | 2.91e+07 | 3.01e+07 | 2.90e+07 | 2.86e+07 | 2.36e+07 | 2.35e+07 |
| Q9QZD8 | GALVTVGQLSCYDQAK | Slc25a10 | 37.93 | 6.50e+07 | 7.03e+07 | 6.25e+07 | 5.04e+07 | 4.89e+07 | 5.35e+07 |
| Q9QZD8 | GLFPAGIR | Slc25a10 | 36.7 | 7.41e+07 | 7.57e+07 | 7.26e+07 | 6.12e+07 | 5.25e+07 | 5.70e+07 |
| P10404 | SLTSLSEVVLQNR | -- | 45.67 | 1.73e+09 | 2.26e+09 | 2.13e+09 | 2.16e+09 | 1.77e+09 | 1.74e+09 |
| P10404 | ISVVQALVLTQQYHQLK | -- | 50.94 | 1.24e+08 | 1.22e+08 | 1.22e+08 | 1.90e+08 | 1.83e+08 | 1.81e+08 |
| P97352 | TLDVNQDSELR | S100a13 | 20.69 | 3.15e+07 | 3.09e+07 | 3.15e+07 | 4.21e+07 | 4.06e+07 | 3.83e+07 |
| P03930 | VSSQTFPLAPSPK | Mtatp8 | 32.31 | 4.33e+07 | 4.37e+07 | 4.34e+07 | 7.95e+07 | 7.67e+07 | 5.99e+07 |
| P03930 | IYLPHSLPQQ | Mtatp8 | 34.18 | 2.91e+07 | 3.01e+07 | 3.40e+07 | 1.09e+07 | 1.02e+07 | 1.03e+07 |
| Q9CXZ1 | DTQLITVDEK | Ndufs4 | 25.07 | 1.11e+07 | 1.51e+07 | 1.35e+07 | 1.30e+07 | 1.15e+07 | 1.19e+07 |
| Q9CXZ1 | LDITTLTGVPEEHIK | Ndufs4 | 43.22 | 1.00e+07 | 1.05e+07 | 1.09e+07 | 1.18e+07 | 1.04e+07 | 1.11e+07 |
| Q60876 | VALGDGVQLPPGDYSTTPGGTLFSTTPGGTR | Eif4ebp1 | 51.36 | 6.25e+05 | 6.14e+05 | 6.07e+05 | 8.77e+05 | 1.01e+06 | 1.09e+06 |
| P26350 | VAEDDEDDDVDTK | Ptma | 8.49 | 3.53e+07 | 3.41e+07 | 3.47e+07 | 6.38e+07 | 5.63e+07 | 5.54e+07 |
| P46414 | VLAQESQDVSGSR | Cdkn1b | 11.46 | 2.36e+07 | 2.52e+07 | 2.51e+07 | 3.96e+07 | 3.93e+07 | 4.19e+07 |
| P46414 | TEENVSDGSPNAGTVEQTPK | Cdkn1b | 15.4 | 6.60e+06 | 6.29e+06 | 6.32e+06 | 1.07e+07 | 1.01e+07 | 1.10e+07 |
| Q9WVS8 | YFLYQLLR | Mapk7 | 52.21 | 1.81e+06 | 1.52e+06 | 1.57e+06 | 1.60e+06 | 1.45e+06 | 1.75e+06 |

C, NSC34-hSOD1G93A cells. S, NSC34-hSOD1G93A cells treated with ET-1.

**Table S3.** Protein Quantification by PRM (S/C)

| **Protein Accession** | **Protein Name** | **Gene**  **Name** | **Ratio (PRM)** | **p-value (PRM)** | **Ratio (Proteomics)** |
| --- | --- | --- | --- | --- | --- |
| O35345 | Importin subunit alpha-7 | Kpna6 | 1.21 | 7.02e-03 | 3.87 |
| P03930 | ATP synthase protein 8 | Mtatp8 | 1.28 | 2.55e-02 | 1.6 |
| P10404 | MLV-related proviral Env polyprotein | -- | 0.85 | 4.72e-02 | 0.65 |
| P10922 | Histone H1.0 | H1-0 | 0.64 | 1.24e-02 | 0.61 |
| P16460 | Argininosuccinate synthase | Ass1 | 1.48 | 4.49e-03 | 1.52 |
| P26350 | Prothymosin alpha | Ptma | 0.59 | 3.40e-04 | 0.63 |
| P46414 | Cyclin-dependent kinase inhibitor 1B | Cdkn1b | 0.61 | 2.19e-05 | 0.61 |
| P97352 | Protein S100-A13 | S100a13 | 0.78 | 8.67e-04 | 0.65 |
| P97450 | ATP synthase-coupling factor 6, mitochondrial | Atp5pf | 0.58 | 6.09e-03 | 0.53 |
| Q60876 | Eukaryotic translation initiation factor 4E-binding protein 1 | Eif4ebp1 | 0.62 | 1.80e-03 | 0.66 |
| Q8BI72 | CDKN2A-interacting protein | Cdkn2aip | 1.2 | 1.76e-02 | 1.63 |
| Q9CQY5 | Magnesium transporter protein 1 | Magt1 | 1.22 | 7.08e-02 | 1.68 |
| Q9QZD8 | Mitochondrial dicarboxylate carrier | Slc25a10 | 1.3 | 2.29e-03 | 4.3 |
| Q3THK7 | GMP synthase [glutamine-hydrolyzing] | Gmps | 1.12 | 7.80e-02 | 1.93 |
| Q9CXZ1 | NADH dehydrogenase [ubiquinone] iron-sulfur protein 4, mitochondrial | Ndufs4 | 1.01 | 8.61e-01 | 1.75 |
| Q9WVS8 | Mitogen-activated protein kinase 7 | Mapk7 | 1.02 | 8.00e-01 | 1.71 |

C, NSC34-hSOD1G93A cells. S, NSC34-hSOD1G93A cells treated with ET-1.
